# Acetylation of Checkpoint suppressor 1 enhances its stability and promotes the progression of triple-negative breast cancer

**DOI:** 10.1038/s41420-022-01269-x

**Published:** 2022-12-01

**Authors:** Zhaowei Xu, Shuyan Liu, Chun Feng, Fuyi Xu, Demin Kong, Jia Mi, Chunhua Yang, Guilong Zhang, Pengfei Wei, Buyan-Ochir Orgil, Jonas Bergquist, Geng Tian

**Affiliations:** 1 Shandong Technology Innovation Center of Molecular Targeting and Intelligent Diagnosis and Treatment, Binzhou Medical University, Yantai, China; 2 School of Pharmacy, Binzhou Medical University, Yantai, China; 3 The Second Medical College, Binzhou Medical University, Yantai, China; 4 University of Tennessee Health Science Center, Memphis, TN USA; 5 Analytical Chemistry and Neurochemistry, Biomedical Centre, Uppsala University, Uppsala, Sweden

**Keywords:** Breast cancer, Acetylation

## Abstract

Checkpoint suppressor 1 (CHES1), a transcriptional regulator, had been dysregulated in many types of malignancies including breast cancer, and its expression level is strongly associated with progression and prognosis of patients. However, the underlying regulatory mechanisms of CHES1 expression in the breast cancer and the effects of post-translational modifications (PTMs) on its functional performance remain to be fully investigated. Herein, we found that CHES1 had a high abundance in triple-negative breast cancer (TNBC) and its expression was tightly associated with malignant phenotype and poor outcomes of patients. Furthermore, we confirmed that CHES1 was an acetylated protein and its dynamic modification was mediated by p300 and HDAC1, and CHES1 acetylation enhanced its stability via decreasing its ubiquitination and degradation, which resulted in the high abundance of CHES1 in TNBC. RNA-seq and functional study revealed that CHES1 facilitated the activation of oncogenic genes and pathways leading to proliferation and metastasis of TNBC. Taken together, this research established a novel regulatory role of acetylation on the stability and activity of CHES1. The results demonstrate the significance of CHES1 acetylation and underlying mechanisms in the progression of TNBC, offering new potential candidate for molecular-targeted therapy in breast cancer.

## Introduction

Breast cancer is the most common malignant carcinoma in women worldwide [1]. Based on the expression difference of specific hormone receptors and genes, breast cancer is mainly classified into five subtypes including luminal A, luminal B, human epidermal growth factor receptor 2 (HER2) + , basal like and normal like [2]. Among them, basal like breast cancer, also named as triple-negative breast cancer (TNBC), exhibits specific pathological characteristics with estrogen receptor alpha (ERα)-, progesterone receptor (PR)- and HER2-, and has more aggressive phenotype and worse prognosis than other subtypes [3]. Due to this feature and heterogeneous tumor microenvironment, there are no effective medicines for patients with TNBC. Therefore, elucidation of the underlying mechanisms in the development and progression of TNBC is critical to identify more reliable bio-markers for clinical diagnose and therapy.

Protein homeostasis is tightly associated with post-translational modifications (PTMs). There are over 200 types of modifications being identified in mammalian cells at present[4]. Acetylation was originally identified as a prevalent PTM for histone, subsequent studies recognized that numerous non-histone proteins could also be modified by acetyl [5]. And acetylation has multiple effects on protein activities, including subcellular location, protein stability, enzymatic activity and transcriptional performance [6, 7]. Additionally, dynamic acetylation and its catalytic enzymes have been shown to be involved in almost all cellular physiological processes, such as energy metabolism, DNA repair, signaling transduction, senescence, immune response, and epigenetic regulation [7]. Notably, the incidence and deterioration of malignant cancers were coupled with abnormal acetylation of proteins due to the fact that acetylation induced activation or repression of cancer-related transcription factors [8]. For example, the first non-histone protein shown to be functionally regulated by acetylation and deacetylation is the tumor suppressor p53 which participates in cell cycle arrest, apoptosis and senescence of tumor cells [9–11]. Similar to the modulation of p53 transcriptional activity by acetylation, acetylation of ERα mediated by p300 regulates its DNA binding affinity and estrogen sensitivity in breast cancer [12, 13]. Consequently, selective inhibitors targeting the specific acetyltransferases and deacetylases have been introduced into clinical treatment of carcinomas [8, 14]. Therefore, uncovering the effect of acetylation on function of the key transcriptional factors and revealing the mechanisms of its regulation in carcinogenesis will provide effective revenue in clinical applications and anti-tumor therapy.

Checkpoint suppressor 1 (CHES1), also named as Forkhead box N3 (FOXN3), is initially identified from yeast and belongs to the FOX protein family as it contains a conserved FOX DNA binding domain in its N-terminal [15]. Subsequent studies revealed that CHES1 served as a transcriptional repressor and mediated histone acetylation *via* its interactions with SIN3A/HDACs complex [16–18]. Chromatin Immunoprecipitation (ChIP)-seq and structural analysis found that CHES1 was a bispecific transcription factor which recognized a canonical forkhead (FKH) motif as well as alternate lower affinity (FHL) motif simultaneously [19, 20]. Functionally, CHES1 modulated many cellular procedures, such as cell cycle arrest [15, 21], embryogenesis [22, 23], glucose metabolism [24], insulin sensitivity [25] and DNA damage response [16]. Moreover, the regulatory role of CHES1 has been established in various malignancies, and its dysfunction has been associated with outcome of patients [26, 27]. In breast carcinoma, CHES1 regulated the proliferation and metastasis of ERα + breast cancer *via* modulating epithelial-mesenchymal transition (EMT) and estrogen signaling pathways [18, 28]. In ERα- and high-grade breast cancer like TNBC, high abundance of CHES1 had been demonstrated [18, 28]. And previous studies reported that the expression of CHES1 was partially regulated by estrogen signaling and miRNAs at the transcriptional and post-transcriptional stages, respectively [28, 29]. However, the detailed mechanism of the post-translational regulation that is responsible for CHES1 overexpression and its function in breast cancer remains to be unclear.

Herein, we investigated the underlying mechanism for the high abundance of CHES1 in TNBC, and revealed the regulatory role of CHES1 in the progression of TNBC. We uncovered that CHES1 was an acetylated protein and the acetylation affected the CHES1 stability and functional performance, resulting in its high abundance and activity in TNBC. This study demonstrated a novel PTM regulatory model of CHES1 that further functionally modulated the oncogenic progression in TNBC.

## Results

### High abundance of CHES1 in TNBC was associated with poor prognosis

**Fig. 1:**
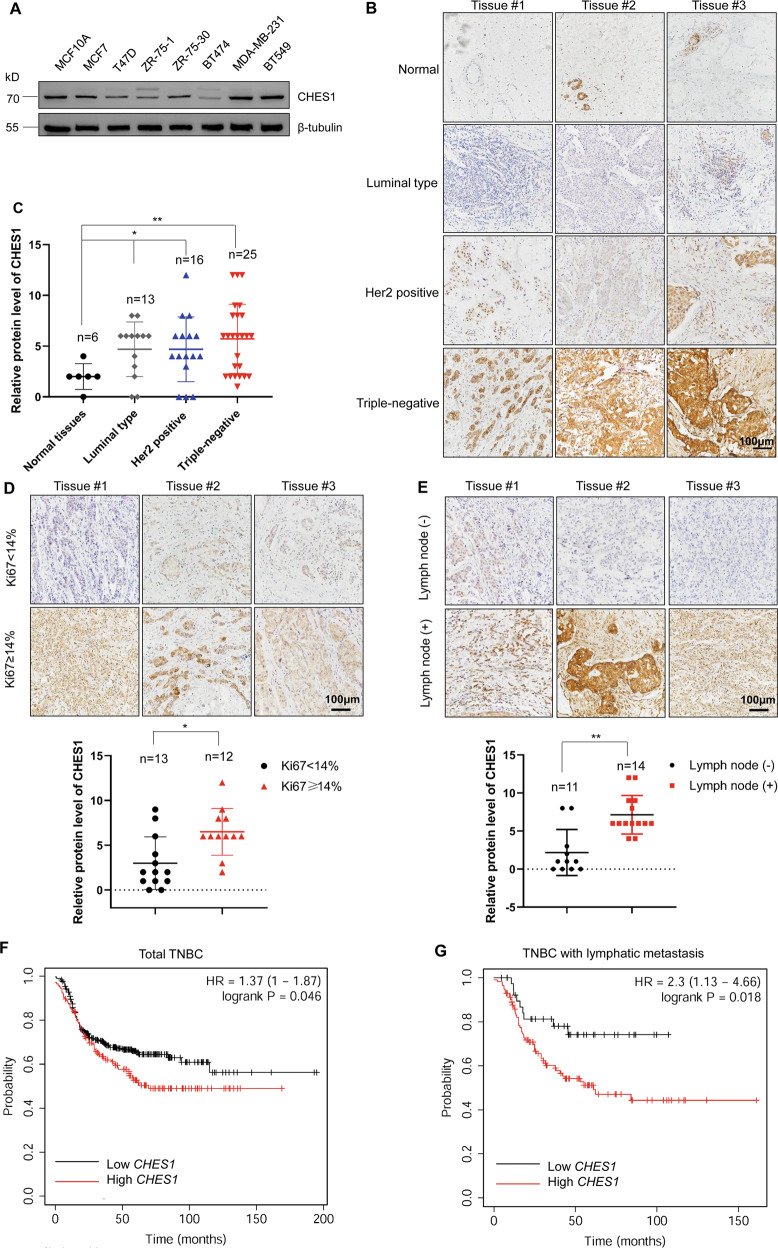
The protein levels of CHES1 in TNBC and its clinical relevance to the prognosis of patients. **A** Western blot detected the protein levels of CHES1 in normal breast epithelial cell and breast cancer cells. **B** Representative images of IHC staining showed the expression pattern of CHES1 in subtypes of breast cancer tissues. **C** The quantified statistics for relative expression of CHES1 in subtypes of breast cancer tissues based on the IHC staining of tissue microarray. *p < 0.05, **p < 0.01. **D** Images of IHC staining showed the expression of CHES1 in TNBC tissues based on the Ki67 intensity. Quantification analysis was showed below. *p < 0.05. **E** Representative images of IHC staining assay showed the expression of CHES1 in TMA based on the lymphatic metastasis status. Quantification analysis were showed below. **p < 0.01. **F**, **G**. Kaplan–Meier curves of relapse-free survival times of total TNBC patients (*n* = 424) (**F**) and breast cancer patients with lymphatic metastasis (*n* = 154) (**G**) stratified by CHES1 expression levels. Data were obtained from the Kaplan–Meier Plotter dataset (http://kmplot.com/analysis/). CHES1 was analyzed with the best probe set (ID:222494_at) with gene chip dataset, the patient with TNBC was restricted by the expression status of ER, PR, and HER2 dectected by IHC or array analysis. Lymph node status was also considered as a restriction condition using the database provided subtype analysis tools. And standard for analysis was kept with default parameters. Statistical significance was determined by the log-rank test.

Considering the dysregulation of CHES1 in carcinomas [26], we determined the levels of CHES1 protein in breast cancer using western blot and tissue microarrays (TMA) analysis. The results showed a significant high-expression of CHES1 in both TNBC cells and tissues (Fig. 1A–C). Furthermore, bioinformatics analysis from UALCAN database (http://ualcan.path.uab.edu/analysis.html) based on the Clinical Proteomic Tumor Analysis Consortium (CPTAC) showed that CHES1 protein progressively increased during breast cancer development (Fig. S1A) [30, 31]. As TNBC exhibits more aggressive phenotype and overexpression of CHES1 compared to other breast cancer subtypes, we focused on the regulatory and prognostic role of CHES1 in TNBC. We firstly analyzed the correlation between CHES1 and Ki67 expression in TNBC tissues and found that tissues with high abundance of CHES1 also had high expression of Ki67, suggesting a potential role of CHES1 in TNBC proliferation (Fig. 1D). In addition, our clinical pathological analysis showed that TNBC patients with lymphatic metastasis had higher expression of CHES1 (Fig. 1E). The association between CHES1 expression and prognosis of TNBC was also evaluated in the Kaplan–Meier Plotter dataset (http://kmplot.com/analysis/) [32], which revealed that TNBC patients with higher CHES1 levels had significantly shorter relapse-free survival time (Fig. 1F), and the high level of CHES1 in these patients with lymphatic metastasis also predicted a poorer prognosis (Fig. 1G). Taken together, high abundance of CHES1 in TNBC positively correlates with more aggressive phenotype and poorer outcome.

### CHES1 maintained the proliferation and invasion of TNBC

**Fig. 2:**
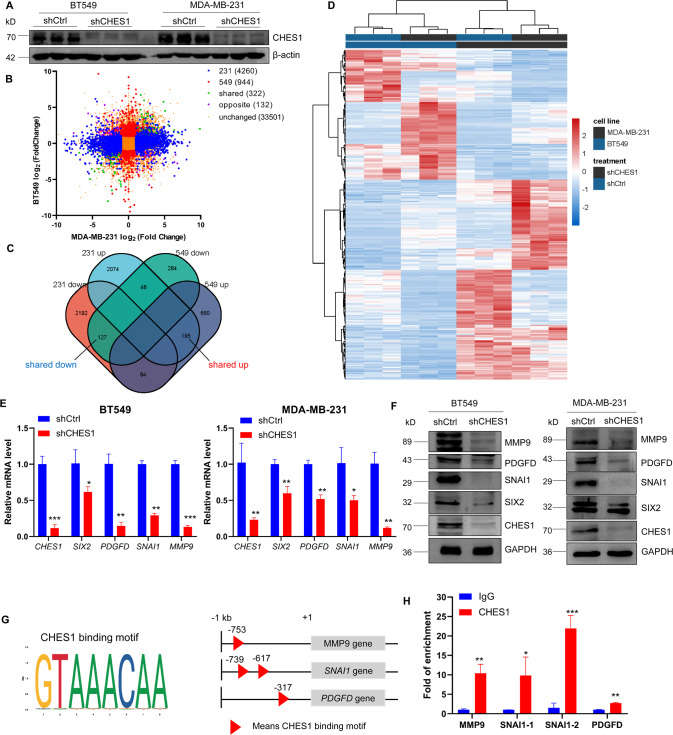
RNA-seq data revealed the genes and pathways associated with CHES1 in TNBC. **A** Western blot showed the knockdown efficiency of shCHES1 in BT549 and MDA-MB-231. **B** Clustering of RNA-seq data in MDA-MB-231 and BT549 with shCHES1. Significance was set based on padj < 0.05 and absolute fold-change > 2. **C** Venn diagram showed that 322 genes shared differentially expressed in both MDA-MB-231 and BT549 cells. **D** Heatmap showed the gene expression pattern of the 322 genes in both cell lines. **E** qPCR assay evaluated the mRNA levels of genes associated with CHES1 in BT549 and MDA-MB-231 cells. *p < 0.05, **p < 0.01, ***p < 0.001. **F** Western blot detected the protein levels of factors associated with CHES1 in BT549 and MDA-MB-231 cells. **G** Schematic representation of the CHES1 binding motif and potential binding sites (red triangle) on the upstream of *MMP9*, *SNAI1*, and *PDGF-D* transcription start site. The data were analyzed and downloaded from the JASPAR dataset (https://jaspar.genereg.net/). **H** The enrichment of CHES1 in the promoter region of genes were evaluated by ChIP. *p < 0.05, **p < 0.01, ***p < 0.001.

Based on the correlation between high abundance of CHES1 and prognostic significance in TNBC, we hypothesized whether CHES1 deficiency has effect on the progression of TNBC. To test this possibility, knockdown of CHES1 was conducted using siRNAs and lentivirus infection in MDA-MB-231 and BT549 cells (Figs. 2A and S1B, C). Then, two types of cells with shCHES1 or shCtrl underwent gene-expression profiling using RNA-sequencing (RNA-seq) followed by bioinformatics analysis to reveal the biological processes associated with CHES1. Differential expression analysis was performed using DESeq2 [33]. Genes with padj <0.05 and fold change >2 found by DESeq2 were defined as differentially expressed genes (DEGs). Overall, clustering of transcriptome sequencing and Venn diagram showed that there were 4260 and 944 DEGs in MDA-MB-231 and BT549 cells as result of CHES1 knockdown, respectively (Fig. 2B, C). Among them, 322 common genes were mutually up- or downregulated in both cell lines (Fig. 2C, D). Gene ontology (GO) and Kyoto Encyclopedia of Genes and Genomes (KEGG) enrichment analysis showed that these 322 genes were involved in multiple pathways associated with breast cancer progression, such as extracellular matrix (ECM) interaction, cell differentiation, DNA replication and signaling transduction (Figs. S2A, B). Gene Set Enrichment Analysis (GSEA) of MDA-MB-231 or BT549 individual data embedded with GO or KEGG also revealed that these DEGs were enriched in signaling pathways including cell cycle, ECM receptor interaction, tight junction and TGF-β signaling (Fig. S2C, D). We next analyzed RNA-seq profiling data to find the downstream target genes regulated by CHES1 and identified many key factors recapitulating the proliferative and metastatic phenotypes in TNBC, including *SIX2*, *SNAI1*, *PDGF-D*, and *MMP9* [34–37]. All these genes were selected to further evaluate their relative mRNA and protein expression in TNBC cells. The results indicated that CHES1 knockdown significantly inhibited these genes expression on the transcription and protein levels (Fig. 2E, F). In addition, ChIP assay confirmed the enrichment of CHES1 on the promoter regions of *SNAI1, PDGF-D, MMP9* but not *SIX2* (Fig. 2G, H), indicating the direct or indirect regulation of CHES1 on the transcriptional expression of these genes. Totally, these results suggested the important role of CHES1 in regulating TNBC progression *via* modulation of the proliferation-related and pro-metastatic genes.

**Fig. 3:**
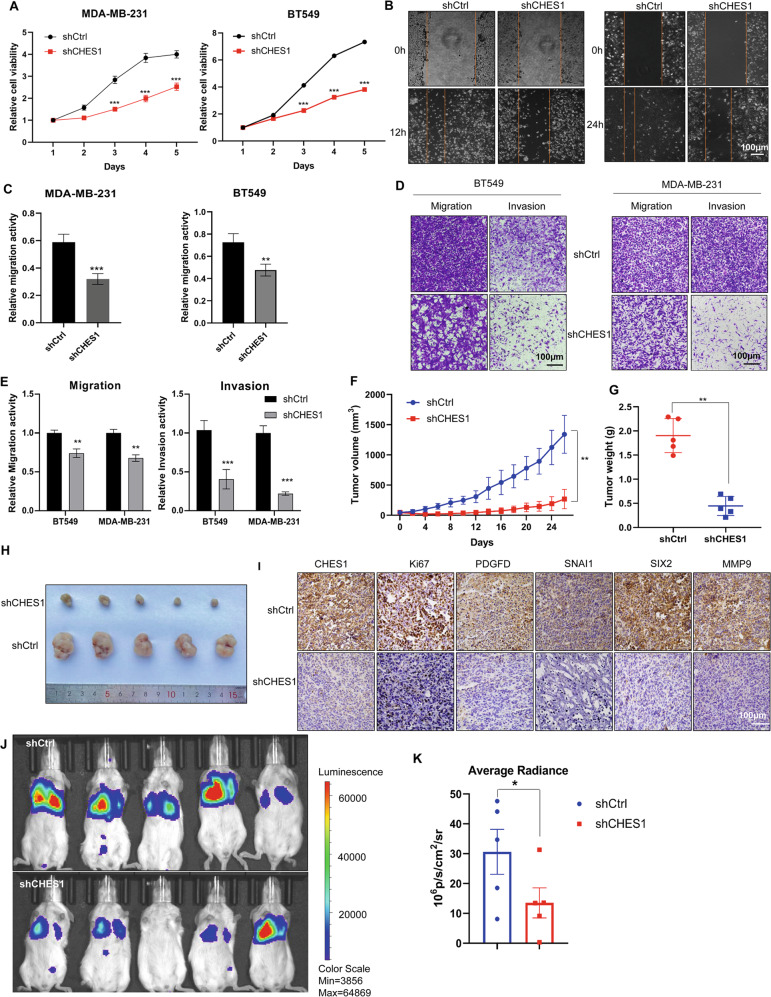
Knockdown of CHES1 impaired the proliferation and invasion of TNBC in vitro and in vivo. **A** CCK8 assay evaluated the effect of shCHES1 on the proliferation of MDA-MB-231 and BT549 cells. ***p < 0.001. **B**, **C** Scratch wound-healing assay showed the effect of shCHES1 on the movement of MDA-MB-231 (left) and BT549 (right) cells. **p < 0.01, ***p < 0.001. **D** Transwell assay determined the effect of shCHES1 on the migration and invasion of MDA-MB-231 and BT549 cells. **E** Colume diagram shown the statistical significance between shCtrl and shCHES1 on the metastasis potential of TNBC. Data were shown means ± SD, **p < 0.01, ***p < 0.001. **F**, **G** Stably knockdown of CHES1 exhibited a significant reduced role in tumor volume (**F**) and tumor weight (**G**) compared to control group (each group *n* = 5). Data of tumor volume were shown with means ± SD, tumor weights were shown with means ± SD, **p < 0.01. **H** The captured image of tumors showed the tumor size of two groups. **I** IHC assays showed the indicated proteins expression in the tumor tissues from two groups. **J** Qualified bioluminescence imaging showed the lung metastasis of shCtrl or shCHES1 groups in tail-vain metastasis model. **K** Histogram showed the average radiance of metastases in two groups. Data were shown the average radiance ±SEM, *p＜0.05.

To further define role of CHES1 in the proliferation and invasion of TNBC, functional experiments were performed in vitro and in vivo. Growth curve monitoring and flow cytometry assays revealed that proliferation inhibition and cell cycle arrest appeared in CHES1-knockdown TNBC cells compared to controls (Figs. 3A and S3A). Scratch wound-healing and Transwell assays found that CHES1 knockdown significantly repressed the migration and invasion of TNBC (Fig. 3B–E), the above results demonstrated that CHES1 maintained the proliferation and invasion of TNBC cells in vitro. Next, we evaluated the potential role of CHES1 in tumorigenesis of TNBC in vivo using human breast cancer xenograft mouse models carrying MDA-MB-231 cells with shCHES1 or shCtrl. The results showed that mice with CHES1 knockdown exhibited smaller tumor volume and weight compared to control (Figs. 3F, H and S3C). And the tumors in shCHES1 group had lower staining of proliferation marker Ki67, along with downregulated expression of PDGF-D, SIX2, SNAI1 and MMP9 (Fig. 3I). Metastatic mouse model using 4T1-luc, murine basal like breast cells with stable luciferase expression, showed that shCHES1 mice had significantly decreased metastases in the lung (Figs. 3J, K and S3D). Collectively, these results demonstrated that CHES1 served as an oncogenic factor to facilitate the growth and metastasis of TNBC in vitro and in vivo.

### CHES1 was acetylated by p300 at multiple lysine residues

**Fig. 4:**
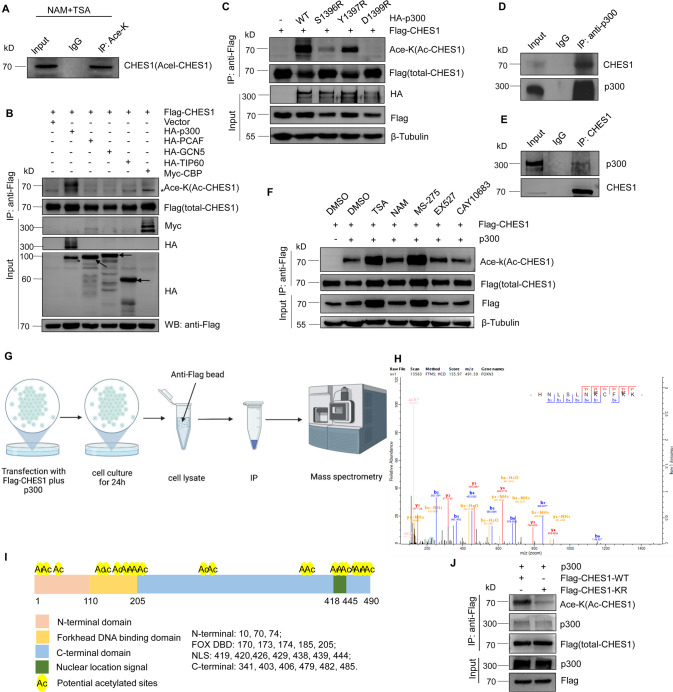
CHES1 was acetylated by p300 and deacetylated by HDAC1. **A** Endogenous IP assay showed the acetylated CHES1 in the presence of pan-HDACs inhibitors. **B** CoIP assay showed that CHES1 could be acetylated by p300 but not other acetyltransferases. “*” denotes the non-specific band. **C** CoIP assay showed the disability of catalytic deficient mutants of p300 on the acetylation of CHES1. **D**, **E** CoIP assay showed the endogenous interaction between CHES1 and p300 in TNBC. **F** IP assay showed the effect of HDACs inhibitors on the acetylation levels of CHES1. **G** The schematic diagram of the main steps for identifying the acetylation lysine sites of CHES1 by mass spectrometry (MS). **H** Tandem mass spectrum of the peptide (m/z = 491.59) HNLSLNKCFKK containing acetylated K170 and K173 of CHES1. The “ac” above the Lys indicates an acetyl remnant on this Lys. **I** The schematic representation of the 21 acetylated lysine sites in the protein domains of CHES1. **J** CoIP assay showed the acetylation levels of wild type CHES1 and its KR mutant.

Previous studies demonstrated that CHES1 regulated genes transcription and epigenetic inheritance *via* interacting with histone deacetylases (HDACs) [18, 28]. As HDACs not only modulate the clearance of histones acetylation, but catalyze deacetylation of non-histone proteins [38]. So we speculated that CHES1 could be acetylated and acetylation further affected the function of CHES1. To test this hypothesis, immunoprecipitation (IP) assay was conducted and the results showed that endogenous CHES1 was acetylated in the presence of pan-HDAC inhibitors trichostatin A (TSA) and nicotinamide (NAM) (Fig. 4A). Next, we screened the acetyltransferases including p300, CBP, TIP60, PCAF and GCN5 to identify the one that is responsible for CHES1 acetylation, the result showed that CHES1 acetylation only could be detected with ectopic expression of p300 but not with others (Figs. 4B and S4A). Moreover, the enzymatic inactive mutations of p300 exhibited attenuated ability in catalyzing CHES1 acetylation (Fig. 4C). Likewise, p300 knockdown and its selective inhibitor C646 largely decreased the acetylation of CHES1 (Fig. S4B, C). Furthermore, the interaction between CHES1 and p300 was confirmed by Co-IP assays in TNBC (Fig. 4D, E). Next, we introduced HDACs inhibitors to identify the enzyme in charge of CHES1 deacetylation and found that TSA (Class I HDACs pan-inhibitor) and MS-275 (HDAC1 specific inhibitor) significantly increased the acetylation of CHES1 (Fig. 4F). These data indicated that HDAC1 was responsible for the deacetylation of CHES1. We also conducted HDAC1 knockdown using siRNAs to confirm the increased CHES1 acetylation (Fig. S4C). Taken together, we demonstrated that CHES1 was an acetylated protein, and this dynamic PTM was mediated by acetyltransferase p300 and deacetylase HDAC1.

In addition, total acetylated CHES1 was purified by IP assay and subjected to mass spectrometry analysis to map the acetylated sites of CHES1 (Fig. 4G, H), the detailed protocol was described previously [39]. The results revealed that CHES1 was acetylated at 21 lysine residues, located in different domains of CHES1 (Fig. 4I). To confirm this result, we created domain truncation or KR mutant (all identified lysines were mutanted to arginines to mimic acetylation deficiency) constructs of CHES1 and performed IP assays. The results showed that truncation of different portions of CHES1 all retained acetylation (Fig. S4D), while KR mutant significantly decreased the acetylation levels and it had weaker interaction with p300 compared to WT CHES1 (Fig. 4J). In conclusion, the above results demonstrated that CHES1 could be acetylated by p300 at multiple lysine residues.

### Acetylation of CHES1 enhanced its stability via decreasing the ubiquitination and degradation

**Fig. 5:**
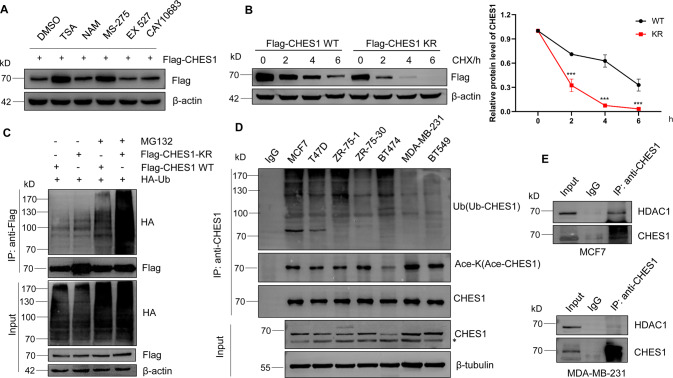
Acetylation of CHES1 enhanced its protein stability via decreasing its ubiquitination. **A** The effect of HDACs inhibitors on the protein levels of exogenous CHES1. **B** Western blot showed the half-life of wild type CHES1 and its KR mutant after treating with 10 μg/ml CHX. The line graph showed the relative intensity of CHES1 protein levels in different time normalized by β-actin, ***p＜0.001. **C** IP assay showed the polyubiquitination levels of wild type CHES1 and its KR mutant with or without treatment of 20 μg/ml MG132 for 6 h. **D** IP assay detected the acetylation and ubiquitination of endogenous CHES1 in different types of breast cancer cells pretreated with TSA and NAM. “*” denotes the non-specific band. **E** CoIP assay showed the endogenous interaction between CHES1 and HDAC1 in MCF7 and MDA-MB-231 cells.

Acetylation has been confirmed to regulate the ubiquitination and stability of target proteins (like SNAI1 and α-tubulin), indicating the crosstalk between PTMs [40, 41]. Similar to that, we also observed that acetylated CHES1 was accompanied with an increase in protein levels (Fig. 4F). To validate the effect of acetylation on CHES1 expression, CHES1 levels were evaluated in response to inhibitors of HDACs and the results showed that TSA and MS-275 significantly increased the exogenous CHES1 protein levels (Fig. 5A). And the effect of HDAC inhibitor on increasing in CHES1 expression was specific as the well-documented acetylated protein p53 had little change with treatment of TSA (Fig. S5A). To further support our speculation, cycloheximide chase and ubiquitination detection assays were performed and the results found that KR mutant had shorter half-life and stronger ubiquitination in the presence of MG132 compared that of WT CHES1 (Fig. 5B, C). Furthermore, we also constructed KQ mutant (all identified lysines were mutanted to glutamines to mimic acetylation condition) to evaluate the acetylation on the stability of CHES1. However, the results showed that KQ mutant only had a little longer half-life than WT, but didn’t exhibit lower ubiquitination modification (Fig. S5C and S5D). The probability accounting for this phenomenon is that KQ mutant just mimic the charge changes of amino acids, and the total effect of acetylation on the protein structure and stability is hard to display, or the acetylation of CHES1 WT containing multiple acetylated sites is sufficient to influence its protein stability and functional performance in cellular physiologic condition, KQ mutant cannot enhance this effect. In totally, these results indicated that acetylation of CHES1 enhanced its stability *via* decreasing its ubiquitination and degradation.

Then we confirmed the expression patterns of CHES1 on transcription or protein levels in different subtypes of breast cancer. We found the *CHES1* mRNA levels determined using quantitative PCR (qPCR) were not totally consistent with CHES1 protein expression in different types of breast cancer cells (Figs. S5B and 1A), it suggested that differential effects of PTMs such as acetylation and ubiquitination on CHES1 stability were associated with our aforementioned results. Specifically, MDA-MB-231 and BT549 cells expressing higher CHES1 protein had higher acetylation, but lower ubiquitination levels (Fig. 5D), this indicated a reversed pattern between acetylation and ubiquitination levels of CHES1 in breast cancer cells. Interestingly, we found no interaction between CHES1 and HDAC1 in TNBC cells but a persistent interaction between CHES1 and p300 (Figs. 5E and S5E, F), which might promote the acetylation and stability of CHES1. Our result was consistent with previous study that CHES1 only interacted with HDAC1 in ERα + breast cancer but not TNBC [18]. In summary, we suggested that CHES1 acetylation might contradict its ubiquitination and enhance its protein stability, and this regulatory model explained the higher abundance of CHES1 seen in TNBC compared to other subtypes of breast cancer.

### Acetylation of CHES1 promotes TNBC progression via gain-of-function

**Fig. 6:**
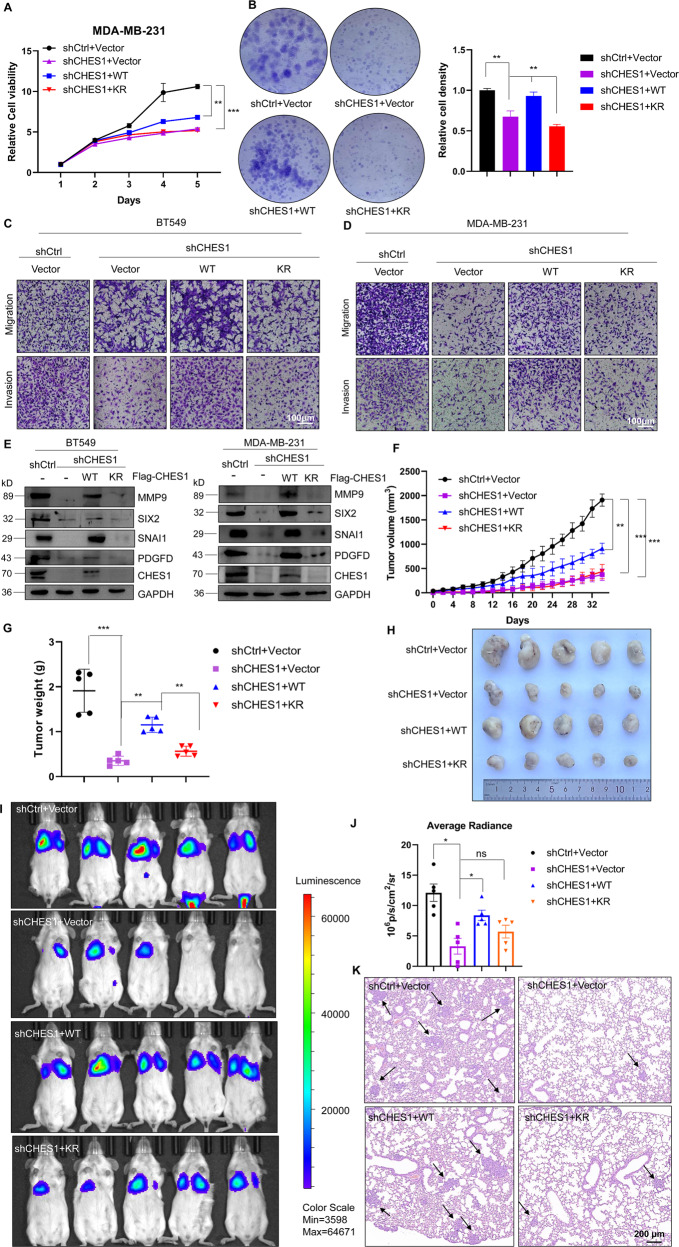
Acetylation of CHES1 modulated the proliferation and invasion of TNBC in vitro and in vivo. **A**, **B** CCK8 and colony formation assays evaluated the effect of shCHES1 with or without overexpression of wild type CHES1 or its KR mutant on the cell viability of MDA-MB-231 cells. The column showed the quantified analysis of relative cell viability from colony formation assay. **p < 0.01, ***p < 0.001. **C**, **D** Transwell assay evaluated the effect of shCHES1 with overexpression of wild type CHES1 and its KR mutant on migration and invasion of MDA-MB-231 and BT549 cells. **E** Western blot showed that the downstream factors regulated by CHES1 could be rescued by overexpression with WT or KR CHES1 in MDA-MB-231 and BT549 cells. **F** Tumor volume showed the growth curve of tumors in vivo from four groups as time extended. Data were means ± SD, **p < 0.01, *** *p* < 0.001. **G** The scatter plot showed the tumor weights removed from four groups at 34th day post injection. **p < 0.01, *** *p* < 0.001. **H** The captured image of tumors showed the tumor size of four groups. **I** Qualified bioluminescence imaging showed the lung metastasis of four groups in tail-vain metastasis model. **J** Histogram showed the average radiance of metastases in two groups. Data were shown the average radiance ±SEM, *p < 0.05, ns means no significant difference. **K** H&E staining showed the metastatic nodes of lungs from four groups. Black arrows indicated the metastatic tumor cells in lung.

As CHES1 acetylation has been strongly associated with its stability, we next determined the effects of acetylation on CHES1 function in TNBC using epistasis assays of proliferation and metastasis, which rescued CHES1 function in response to CHES1 knockdown by shCHES1 followed by overexpression of WT or KR CHES1, respectively. Growth curves demonstrated that overexpression of WT CHES1 rescued the inhibition effect of shCHES1 on the proliferation of MDA-MB-231, while KR CHES1 had no significant effect on cell growth (Fig. 6A). Colony formation assay showed that shCHES1 repressed the number and size of colonies, whereas WT CHES1 overexpression recovered the colony formation, but not KR mutant (Fig. 6B). Likewise, Transwell assay found that WT CHES1 ectopic expression following shCHES1 knockdown effectively rescued the invasion and migration of TNBC cells, while KR CHES1 failed to enhance the aggressive potential of TNBC (Fig. 6C, D). We also tested the effect of KQ mutant on the proliferation and migration of BT549, but the results indicated that it could not rescue the cell growth and motivation like WT (Fig. S6A, B), so the following functional investigation mainly focused the comparison between WT and KR CHES1. Consistently, expression of genes regulated by CHES1 remained to be recovered with WT overexpression in TNBC cells in vitro (Fig. 6E), suggesting that acetylation of CHES1 may regulate the proliferation and invasion of TNBC *via* facilitating the functional performance of CHES1. Furthermore, mouse models with human breast cancer xenograft were utilized to further explore the effect of CHES1 acetylation on the tumorigenesis of TNBC. Knockdown of CHES1 significantly repressed the growth of MDA-MB-231 in vivo, followed by partial rescue of the tumorigenesis potential of TNBC and no or minimal rescue in response to force expression of WT CHES1 and KR substitution, respectively (Figs. 6F–H and S6C). In addition, tumor section staining for Ki67 and CHES1-regulated genes found that WT CHES1, but not KR mutant, increased the proliferative potential and partially reversed the phenotypic inhibition of CHES1 knockdown (Fig. S6E, F). Specifically, tail-vein metastasis assay and quantitative bioluminescence imaging showed that overexpression of WT CHES1 following with shCHES1 regained the comparative metastasis potential compared to the shCtrl group, while KR mutant had weaker enhancing effect on the metastatic potential of 4T1 in vivo (Figs. 6I, J and S6D). Pathological analysis by H&E staining of lung in mice revealed that most metastatic lesions were detected in shCtrl groups, followed by shCHES1 + WT overexpression, and shCHES1 + KR mutant, whereas shCHES1 mice exhibited minimal metastatic events (Fig. 6K). In summary, we showed that acetylation of CHES1 mediated tumorigenesis and metastasis of TNBC via gain-of-function in vitro and in vivo.

## Discussion

Dysregulation of CHES1 was found in multiple types of malignancies, but the detailed mechanisms of regulation for CHES1 and its abundance remain unclear [26]. In this study, we revealed that the mRNA levels of *CHES1* were inconsistent with its protein expression pattern in breast cancer cells (Figs. 1A and S5B). Therefore, we speculated the PTM might regulate CHES1 abundance in breast cancer. Previous studies found the interactions between CHES1 and HDAC1/2 in ERα + breast cancer [28, 38], so we hypothesized that non-histone CHES1 might undergo acetylation and the acetylation further affected its expression. Subsequent results validated that CHES1 could be acetylated and this dynamic PTMs were regulated by p300 and HDAC1 (Fig. 4B, F). Furthermore, CHES1 acetylation modulated its protein stability and abundance *via* reducing its ubiquitination with subsequent decrease in protein degradation (Fig. 5C). Based on these results, we conclude that CHES1 is an acetylated protein and its regulatory crosstalk between acetylation and ubiquitination causes different levels of CHES1 in different subtypes of breast cancer. Consistently, we observe that stronger acetylated CHES1 is associated with higher CHES1 expression in TNBC compared to other types of breast cancer cells (Fig. 5D). We suggest that the significance of CHES1 in the progression of TNBC is based on its acetylation and the absence of interaction between CHES1 and HDAC1 in TNBC (Fig. 5E). However, the acetylation of CHES1 and its detailed regulatory model in other types of tumors deserves further investigation.

In addition, our present research revealed the significance of CHES1 in the progression of TNBC. Previous studies by us and Li et al. reported that CHES1 regulated proliferation and metastasis in ERα + breast cancer, and ERα- breast cancer had higher expression of CHES1 compared to other breast cancer subtypes [18, 28]. This fact raised our interest to explore the expression patterns of CHES1 and define its functional role in other subtypes of breast cancer, such as TNBC. First, we determined CHES1 protein expression in TNBC by clinicopathologic and bioinformatics analysis. The results demonstrated an upregulation of CHES1 in TNBC and its overexpression was closely associated with poor prognosis of patients. Thus, we performed series of functional experiments to validate the function of CHES1 in TNBC and found CHES1 may serve as an oncogenic factor governing the proliferation and invasion of TNBC. Based on further gene expression profiling and clustering enhancement analysis, CHES1 has been associated to genes such as *SIX2*, *MMP9*, *SNAI1*, and *PDGFD*, which are involved in cell growth, focal conjunction, ECM receptor interaction and the associated pathways largely modulate the tumorigenesis and invasion of breast cancer [37]. We suggested that the oncogenic role of CHES1 in sustaining the proliferation and metastasis of TNBC was executed *via* regulating these genes mentioned above. Notably, this finding was partially consistent with the study reported by Li et al. [18], while seemingly confliced with our previous report that CHES1 inhibited the tumorigenesis of ERα + breast cancer *via* regulating the activity of ERα [28]. An alternative possibility is that CHES1 serves as a transcription factor and its functional performance is largely dependent on the signaling stimulation and interactive network in specific tumor cellular environment, the lack of interaction between CHES1 and ERα in TNBC may account for its specific regulation in TNBC. We also speculate that CHES1 function may be naturalized to facilitate progression of TNBC with the deterioration of cancer stage and signaling crosstalk. In addition, our RNA-seq data and subsequent assays indicated that CHES1 maintained the oncogenes expression in TNBC, which also conflicted with its role as a transcription repressor. Nevertheless, the interaction between CHES1 with transcriptional activator p300 in breast cancer cells might partially explain its dual function in transcriptional regulation (Figs. 4D, E and S5F), Yu et al. also found that CHES1 served as a transcriptional activator to upregulate *Dpp* expression in Drosophila testis [42]. Therefore, CHES1 may serve as an activator or repressor in transcriptional regulation that may be dependent on a tissue specificity or co-factor it interacted with, which warrant further deep investigation on this issue. Another limitation of this study was that the main acetylated sites of CHES1 has not been identified based on the present data. IP assay using the truncated mutant of CHES1 indicated that Forkhead DBD domain might have higher acetylation compared with other domains (Fig. S4D), this suggested the acetylation might affect its affinity with DNA. In addition, the KQ mutant to mimic acetylation of CHES1 also didn’t exhibit its expected effect on the stability and functional performance of CHES1 (Figs. S5C, D and S6A, B). On the one hand, the activity of CHES1, with multiple acetylated residues identified by MS, was closely associated with its acetylation in the physiological conditions, it might have relatively high acetylation levels in TNBC, so the KQ is hard to enhance its function anymore. On the other hand, the KQ mutant just mimic the charge changes of amino acids caused by acetylation, but couldn’t totally replace the structural alternation or protein activity in the cellular environment.

Aberrant reprogramming of energy metabolism was frequently detected in many types of carcinomas including breast cancer, and CHES1 has been reported to modulate glucose homeostasis and insulin sensitivity in zebrafish and mouse models [24, 25, 43]. Consistently, our transcriptomics data also found CHES1 being involved in specific metabolic pathways of TNBC, such as unsaturated fatty acid biosynthesis and fatty acid elongation (Fig. S2A, B). The proliferation and pro-metastatic phenotype in TNBC has been shown to be tightly coupled with specific metabolic flux, and abnormal acetylation is also associated with energetic metabolism [44]. Therefore, more comprehensive research on how CHES1 and whether acetylation-mediated functional modification of CHES1 regulates energetic metabolism of TNBC will aid to understand the metabolic contributions to the progression of breast cancer.

**Fig. 7:**
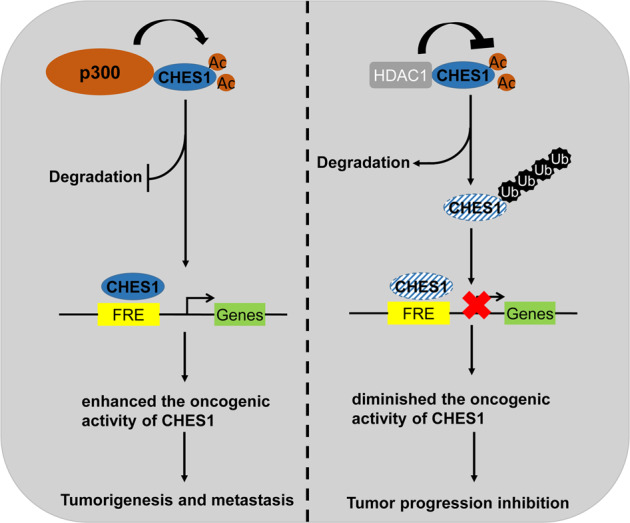
The schematic diagram of the regulatory mechanism of CHES1 acetylation on the progression of breast cancer. The dynamic acetylation of CHES1 mediated by p300 and HDAC1 regulated the tumorigenesis and invasion of breast cancer cells through modulating the stability and transcriptional activity of CHES1.

In conclusion, our study established a regulatory mechanism of acetylation on the stability and functional performance of CHES1 in breast cancer, and highlighted the significance of CHES1 in the tumorigenesis and metastasis of TNBC (Fig. 7). It provided novel insight into the molecular mechanism of CHES1 acetylation in breast cancer and permitted further target therapy for TNBC.

## Material and methods

### Cell culture

MCF7, T47D, ZR-75-1, ZR-75-30, and MDA-MB-231 cell lines were mentioned as described in our previous study [28]. BT474 and BT549 were purchased from iCell Bioscience Inc (Shanghai, China). They were cultured in RPMI-1640 complete medium containing 10% FBS, 100 U/ml penicillin and 0.1 mg/ml streptomycin. Human breast epithelial cells MCF10A were cultured in DMEM/F12 medium containing 5% horse serum, 20 ng/ml EGF, 10 μg/ml insulin, 0.5 μg/ml hydrocortisone, 100 ng/ml cholera toxin, and penicillin-streptomycin. Murine breast cancer cells 4T1 stably expressing luciferase was cultured in RPMI-1640 complete medium.

### Plasmids

Plpc-3×Flag-CHES1, GFP-CHES1 WT and its truncated contructs, p300 and control vector were described previously [28]. HA-PCAF, HA-TIP60, HA-GCN5, HA-p300 WT, S1396R, S1397R, and D1399R mutants were mentioned in our previous studies [45]. pcDNA3.1-Flag-CHES1, KQ and KR mutants were constructed by HedgehogBio Ltd (Shanghai, China).

### Reagents and antibodies

Antibodies against CHES1/FOXN3 (ab129453) and PDGF-D (ab181845) were purchased from Abcam (UK). Antibodies Acetylated-lysine (#9441), p300 (#86377), MMP9 (#13667), and SNAI1 (#3895) were obtained from Cell Signaling Technology (USA). Rabbit polyclonal antibody against SIX2 (WL04402) was purchased from Wanleibio (CN). Flag-M2 mouse monoclonal antibody (F9291) was obtained from Sigma-Aldrich (USA). Flag-tag (20543-1-AP), HA-tag (51064-2-AP) and ubiquitin (10201-2-AP) antibodies were purchased from Proteintech (USA). GFP (GTX113617), HDAC1 (GTX100513) were obtained from GeneTex. CHES1/FOXN3 recombinant polyclonal antibody (1HCLC, #711585) were obtained from Invitrogen (USA). GAPDH (D190090) antibody were purchased from Sangon Biotech (CN). β-tubulin (T0023) and β-actin (T0022) antibodies were purchased from Affinity Biosciences (USA).

Class I HDACs inhibitor Trichostatin A (TSA) (S1045), specific inhibitor for HDAC1 (Entinostat/MS-275, S1053), HDAC2 (CAY10683, S7595), p300 (C646, S7152), p300/CBP (SGC-CBP30, S7256), Puromycin (S7417), and proteasome inhibitor (MG132, S2619) were purchased from Selleck (USA). Protein synthesis inhibitor Cyclohexane (CHX) were obtained from MCE (USA). Class III inhibitor NAM (S1761) and SIRT1 inhibitor Ex527 (SC0281) were obtained from Beyotime BioTech (CN).

### Immunoblots and co-immunoprecipitation

Western blot and Immunoprecipitation (IP) protocols were described previously [28]. Briefly, the cells were washed with cold PBS buffer and then subjected to total protein extraction with Lysis buffer (P0013, Beyotime) mixed with protease inhibitor cocktail (B14001, Biomake). After centrifugation with 12, 000 rpm for 10 min, the suspension was collected for SDS-PAGE or IP. Flag-affinity magnetic beads (B26101, Biomake), Flag-affinity gel (B23101, Biomake), GFP-selector (2-9131-020, IBA Life Sciences) and Protein A&G (B23201, Biomake) magnetic beads were used for exogenous or endogenous IP assays according to the manufacturer’s instruction.

### Transfection and lentivirus infection

Transfection or infection assays were performed when the cells grew to 60–80% confluency. The plasmids or siRNA were transfected into cells using jetPRIME Transfection Kit (114-01, Polyplus) according to manufacturer’s instruction. Lentiviruses expressing Flag-CHES1 WT, Flag-CHES1 KR, and shCHES1 were constructed by OBiO Technology (Shanghai). The concentrated lentivirus supernatant was added to cell culture for infection, 1 μg/ml puromycin was added to select the stable cell as mass pools.

### Mass spectrometry and mapping the acetylation sites of CHES1

The detailed method has been described previously [39]. To detect the acetylated sites of CHES1 in vitro, HEK293T cells were transfected with Flag-CHES1 with or without p300 using polyetherimide (#23966, Polysciences). 48 h post transfection, 1 μM TSA and 5 mM NAM were added for 12 h to block the activity of HDACs. Then the cells were lysed and subjected to immunoprecipitation using Flag-affinity gel. The immunoprecipitate was separated by SDS-PAGE and the gel was staining with Coomassie Blue Staining Solution (P0017F, Beyotime). The positive bands of CHES1 were collected and subjected to in gel digestion, then reversed phase microcapillary/tandem mass spectrometry (LC/MS/MS) was conducted to determine the acetylation sites of CHES1. The detailed acetylated sites of CHES1 were provided at Supplementary table 2. The MS technical service was provided by Bioprofile BioTech (Shanghai).

### Cell proliferation and colony formation assays

The growth curves of cells were measured using Cell Counting Kit-8 (CCK-8, B34302, Biomake). The procedure of cell proliferation and colony formation assays were described in previous study [28]. The cell colony was stained with crystal violet and the absorbance of the crystal violet solution de-stained by 4 % acetic acid was measured at 590 nm to qualify the relative cell density.

### Immunofluorescence and Immunohistochemical staining assays

IF and IHC assays were carried out as described previously [28]. The breast cancer tissue microarray (#HBreD080CS01) was purchased from Shanghai Outdo Biotech Company. The images of staining were captured using Nikon fluorescence microscope system. The scores of IHC staining (0 to 12) were conducted according to the percentage of positively stained tumor cells and staining intensity.

### RNA isolation and qPCR

Total RNA was extracted using RNAiso Plus (9108, Takara), the procedure was described previously [28]. cDNAs were obtained by reverse transcription reaction using PrimeScript™ RT reagent Kit with gDNA Eraser (RR047A, Takara). qPCR assay was conducted to evaluate the relative expression of genes by TB Green® Premix Ex Taq™ II (RR82LR, Takara) with Thermal Cycler Dice™ Real Time System III (TP970, Takara). The specific primers for targeted genes were listed in Supplementary table 1.

### Transwell and scratch wound-healing assays

In order to eliminate the proliferation effect of breast cancer cells, MDA-MB-231 and BT549 cells were subjected to starvation for 24 h with culture medium containing 2% FBS, and then Transwell and scratch wound-healing assays were conducted to evaluate the potential of migration and metastasis. The apical chambers for invasion assay were pre-packaged with Matrigel Matrix (#356230, Corning). 2×10^4^ and 5×10^4^ cells/100 μl were seeded in apical chambers with minimum medium for migration and invasion assays respectively, and basolateral chambers were incubated with complete culture medium. The cells were staining with crystal violet, and the images were captured post 24 h after seeding.

### Mouse xenograft model and metastasis model

All animal experiments were performed according to the regulation set by the Ethics Committee for Biology and Medical Science of Binzhou Medical University. BALB/c mice and athymic nude (BALB/c-null) mice were purchased from GemPharmatech Inc (Nanjing, China) and fed in SPF Animal Center of Binzhou Medical University. The animals were divided into groups based on the body weight and each group has at least five animals. Human breast cancer xenograft model was constructed according to the procedure described previously [28]. Briefly, 5 × 10^6^ stably transfected MDA-MB-231 cells suspended in 100 μl of PBS (mixed with Matrigel at 1:1 ratio) were injected subcutaneously into groin near the 4th mammary gland of mouse. 2 weeks post injection, the body weight and tumor volume were monitored every 2 days. When the biggest tumor volume reached 2,000 mm^3^, the mice were sacrificed and tumors were removed, photographed and weighed. Tail-vein metastasis model were carried out as described previously [46, 47], 5 × 10^5^ 4T1-luc murine breast cancer cells in 100 μl PBS buffer were intravenously injected into the tail vein of the 6 weeks female BALB/c mice, the body weight of each group was measured every 2 days after injection. 7 days post injection, mice were subjected to the intraperitoneal injection of D-luciferin (BD Pharmingen, #556888) solution in D-PBS (Solarbio, #D1040) buffer at 150 mg Luciferin/kg body weight, the firefly luciferase bioluminescence signals of each groups were analyzed by IVIS®Spectrum CT imaging system (PerkinElmer). Then the mice were subjected to euthanasia and the lungs were fixed, the hematoxylin and eosin (H&E) staining was conducted to analyze the metastatic node in lungs.

### Chromatin Immunoprecipitation

ChIP assays were performed as previously described [28]. The primers for the ChIP assay were listed in Supplementary table 1.

### RNA-seq and bioinformatics analysis

The RNA-seq libraries were prepared and sequenced on Illumina Hiseq2000 by Bioprofile Inc (Shanghai, China). The raw reads underwent the following filter to produce clean data: (1) Remove reads containing adapters, (2) remove bases containing non-AGCT at the 5′ end before trimming, (3) trim the ends of reads with low sequencing quality (<Q20), (4) Remove reads containing *N* > 10%, and (5) discard reads with less than 25 bp in length. The clean reads were aligned to the reference genome with HISAT2 [48], followed by quantification with stringtie [49]. Differential expression analysis was performed using DESeq2 [33]. Genes with padj < 0.05 and fold change > 2 found by DESeq2 were defined as differentially expressed genes. Both Human reference genome (GRCh38) and gene model annotation (release-94) were downloaded from the Ensembl (https://useast.ensembl.org/). The sequencing data could be reached in NCBI Short Read Archive (SRA; http://www.ncbi.nlm.nih.gov/sra) under accession number PRJNA809162. Expression of CHES1 protein in breast cancer was analyzed from UALCAN (http://ualcan.path.uab.edu/) based on CPTAC datasets. The Kaplan–Meier plot survival data of breast cancer were obtained from Kaplan–Meier Plotter (http://kmplot.com/analysis/). The CHES1 binding motif and potential binding sites (red triangle) on the transcription start site upstream of MMP9, SNAI1 and PDGFD. The data were analyzed and downloaded from the JASPAR dataset (https://jaspar.genereg.net/) [50]. The heatmap, venn diagrams, GO and KEGG analysis of RNA-seq were performed in Hiplot (https://hiplot.org), a comprehensive and easy-to-use web service for boosting the publication-ready biomedical data visualization [51].

### Statistical analysis

Statistical analysis was performed by GraphPad Prism 8 (GraphPad Software). Data were presented as mean ± SD or SEM with the results of three independent experiments, Student’s *t* test (unpaired, two-tailed) was used to compare two groups of independent samples. *p* < 0.05 was considered statistically significant. *p < 0.05, **p < 0.01, ***p < 0.001, ns means no significant difference.

## Supplementary information

Supplementary Figures

Supplementary table 1

Supplementary table 2

Original Data File

## Data Availability

Raw and processed data of RNA-seq from this study were deposited in the NCBI SRA under accession number PRJNA809162. The full length uncropped original western blots and other original data or materials used in the current study are available in supplementary materials or from the corresponding author on reasonable request.
