# Supplementary material for: Acetylation of Checkpoint suppressor 1 enhances its stability and promotes the progression of triple-negative breast cancer
Source: Cell Death Discov. 2022 Dec 1;8:474. doi: 10.1038/s41420-022-01269-x (PMC9712368; doi:10.1038/s41420-022-01269-x)
Supplement: Supplementary file 1 — Supplementary Figures [file 41420_2022_1269_MOESM1_ESM.pdf]

Supplementary Figure 1.

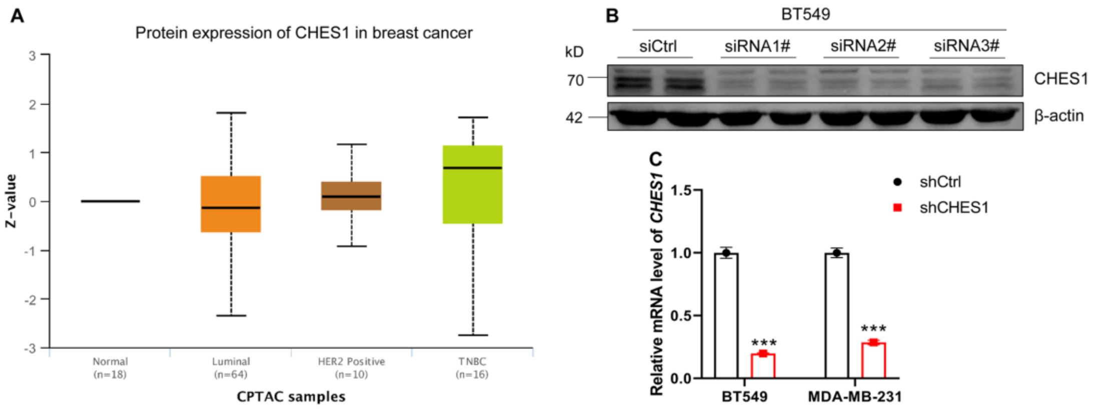

A. Box plots compared the relative protein levels of CHES1 in major subtypes of breast cancer. The results were analyzed from public datasets UALCAN (http://ualcan.path.uab.edu/) based on the data of CPTAC[33]. B. Western blot evaluated the knockdown efficiency of siRNAs on the CHES1 protein in BT549 cells. C. qPCR assay determined the knockdown efficiency of shCHES1 lenti-virus on the mRNA levels of CHES1 in BT549 and MDA-MB-231 cells. ***$p < 0.001$.

Supplementary Figure 2.

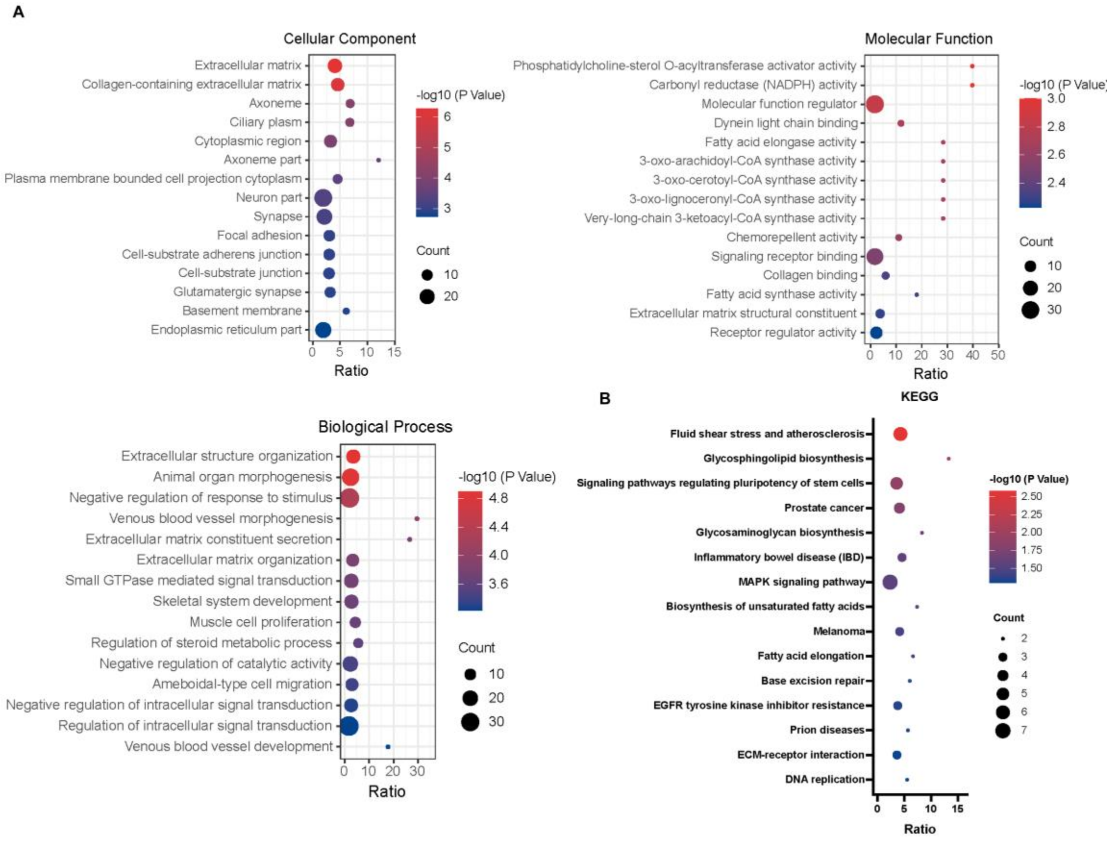

A. GO enrichment analysis showed the enriched biological processes, cellular components and molecular functions of 322 shared genes in MDA-MB-231 and BT549.

B. Gene enrichment analysis based on KEGG revealed the pathways associated with shCHES1 in TNBC.

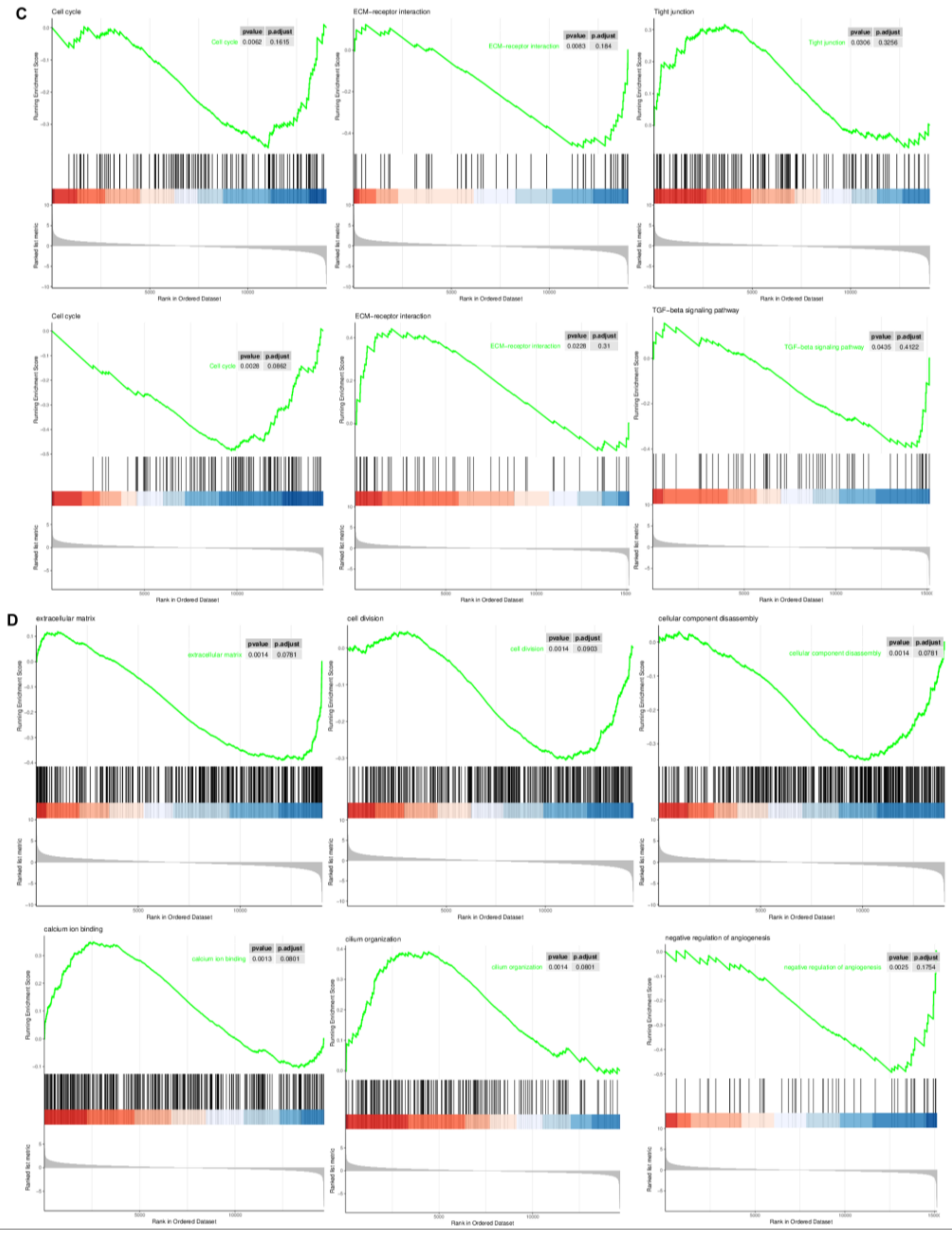

C. GESA with embedded KEGG based on the individual data of MDA-MB-231 (upper row) or BT549 (lower row) revealed the enriched pathways associated with the gene cluster regulated by CHES1. D. GESA with embedded GO based on the individual data of MDA-MB-231 (upper row) or BT549 (lower row) revealed the enriched biological processes in TNBC.

Supplementary Figure 3.

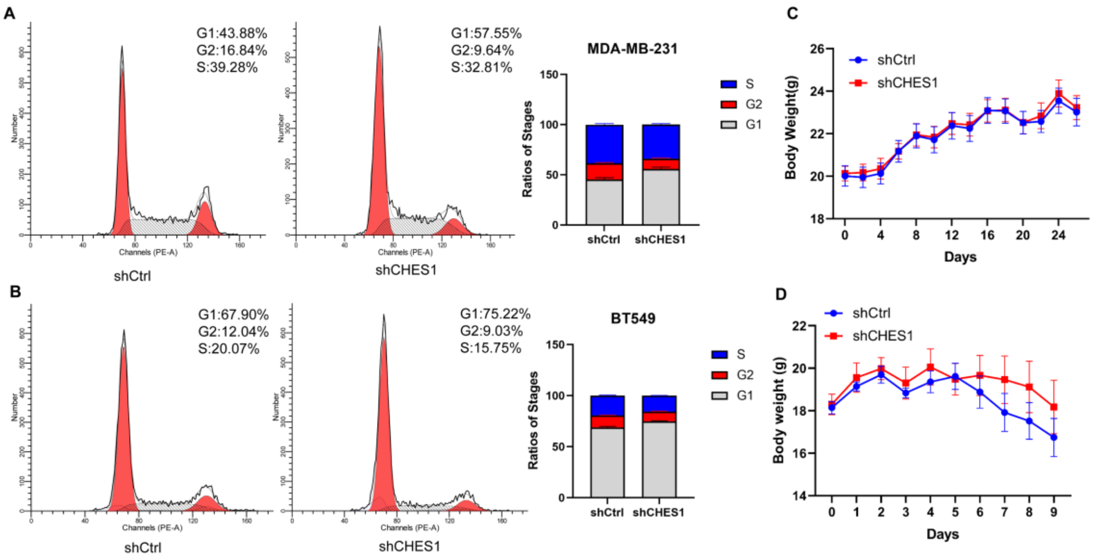

A and B. Flow cytometry determined the effect of shCHES1 on the cell cycle progression of MDA-MB-231 and BT549 cells. C and D. The body weight of two groups mice from shCtrl and shCHES1 for tumorigenesis (C) and metastasis (D) models, respectively.

Supplementary Figure 4.

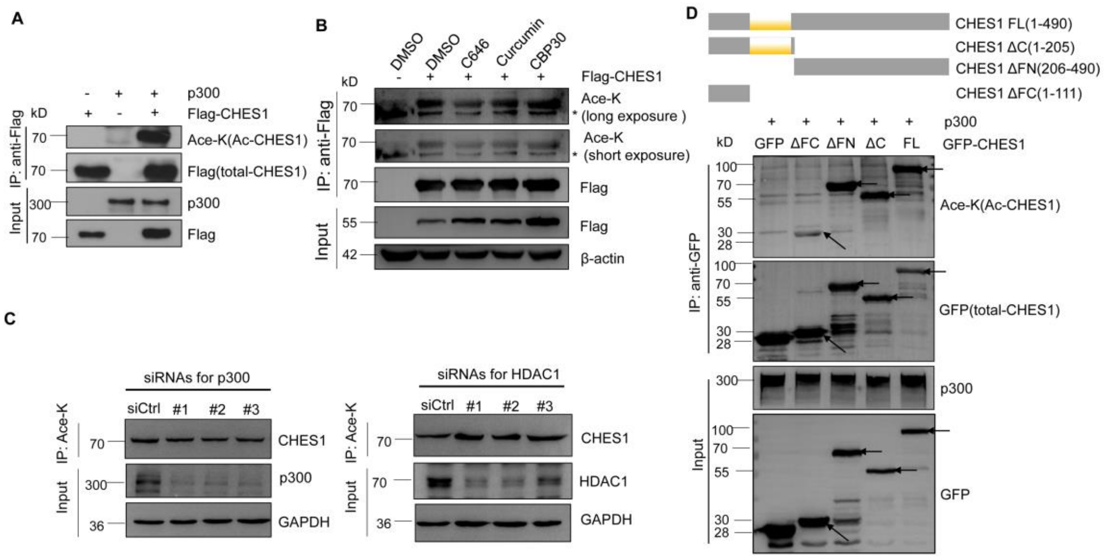

A. IP assay showed the acetylation of exogenous CHES1 mediated by p300. B. IP assay showed the effect of C646, curcumin and CBP30 on the acetylation of CHES1. "*" denotes the nonspecific band. C. IP assays showed the effect of p300 and knockdown with siRNAs on the acetylation of CHES1. D. Wild type and truncated mutants of GFP-CHES1 were used to detect the acetylation of CHES1.

Supplementary Figure 5.

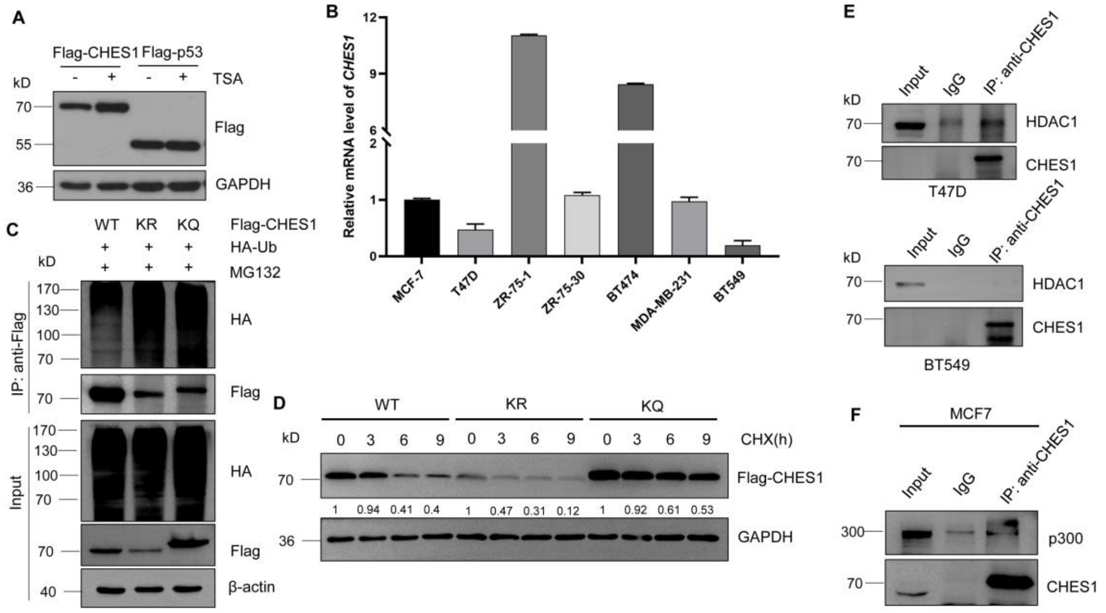

A. Western blot assay evaluated the effect of TSA treatment on the exogenous expression of CHES1 and p53. B. qPCR assay tested the mRNA levels of *CHES1* in different breast cancer cells. C. IP assay evaluated the ubiquitination of WT, KR and KQ CHES1. D. Western blot determined the half-life of WT, KR and KQ with CHX treatment. E. CoIP assay detected the interaction between CHES1 and HDAC1 in T47D and BT549 cells. F. CoIP assay showed the interaction between CHES1 and p300 in MCF7.

Supplementary Figure 6.

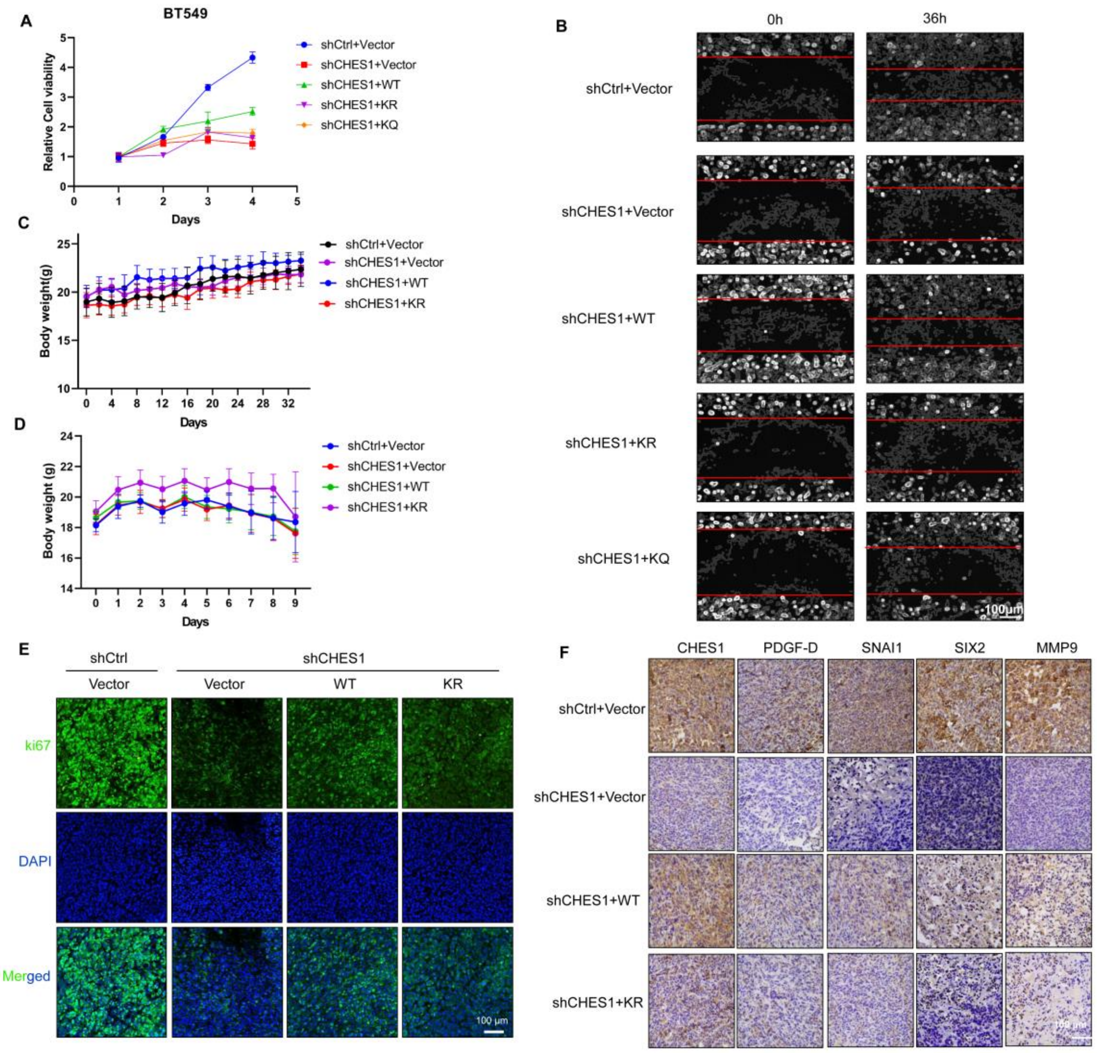

A. CCK8 assay evaluated the rescued effect of WT, KR and KQ on the proliferation potential of BT549 after CHES1 knockdown. B. Scratch wound-healing assay tested the rescued role of WT, KR and KQ on the migration of BT549 after CHES1 knockdown. C and D. The mice weight of four groups for tumorigenesis and tail vain metastasis model, respectively. E. Ki67 staining of tumors from the indicated four groups. F. IHC staining evaluated the expression of PDGFD, SNAI1, SIX2 and MMP9 in the tumor tissues from four indicated groups.
