# Supplementary material for: Acetylation of Checkpoint suppressor 1 enhances its stability and promotes the progression of triple-negative breast cancer
Source: Cell Death Discov. 2022 Dec 1;8:474. doi: 10.1038/s41420-022-01269-x (PMC9712368; doi:10.1038/s41420-022-01269-x)
Supplement: Supplementary file 2 — Supplementary table 1 [file 41420_2022_1269_MOESM2_ESM.docx]

|  | Genes | Primers |
| --- | --- | --- |
| qPCR | *MMP9* | 5’-GGGACGCAGACATCGTCATC-3’ |
|  |  | 5’-TCGTCATCGTCGAAATGGGC-3’ |
|  | *SNAI1* | 5’-ACTGCAACAAGGAATACCTCAG-3’ |
|  |  | 5’-GCACTGGTACTTCTTGACATCTG-3’ |
|  | *CHES1* | 5’-AAATGGAGCGCGGGTCCTGAG-3’ |
|  |  | 5’-GCAGCTGGTGATGCCATTCCT-3’ |
|  | *SIX2* | 5’-AAGGCACACTACATCGAGGC-3’ |
|  |  | 5’-CACGCTGCGACTCTTTTCC-3’ |
|  | *Tubulin* | 5’-CCAAGCTGGAGTTCTCTA-3’ |
|  |  | 5’-CAATCAGAGTGCTCCAGG-3’ |
|  | *PDGFD* | 5’-TCAAGTCCGATGACTACTTTGTG -3’ |
|  |  | 5’-GTGACAGATTCCCAGTTGGTC -3 |
| ChIP | SNAI1-1 | 5’- AGGAGTTACTCTGAAGCAGTTGCCA-3’ |
|  |  | 5’- GGTCCAGTGAGGGAGACAGACGAAG-3’ |
|  | SNAI1-2 | 5’-TGTTTACTTCGTCTGTCTCCCTCAC-3’ |
|  |  | 5’- GTGGCTCTCGGCGGCTTGAAAT-3’ |
|  | MMP9 | 5’- GCCTTAGTAATTAAAACCAATCACC-3’ |
|  |  | 5’- ACAGTAGCAGCCTCTAGAAAACA -3’ |
|  | PDGFD | 5’-CCCTGTCCCCACATCCCACCACCAT-3’ |
|  |  | 5’-CGGGCCTAAACCTGCTCTGCCACTG-3’ |

Supplementary Table 1. The primers information of target genes used in qPCR or ChIP assays.
