# Supplementary material for: Acetylation of Checkpoint suppressor 1 enhances its stability and promotes the progression of triple-negative breast cancer
Source: Cell Death Discov. 2022 Dec 1;8:474. doi: 10.1038/s41420-022-01269-x (PMC9712368; doi:10.1038/s41420-022-01269-x)

Fig 1A

CHES1

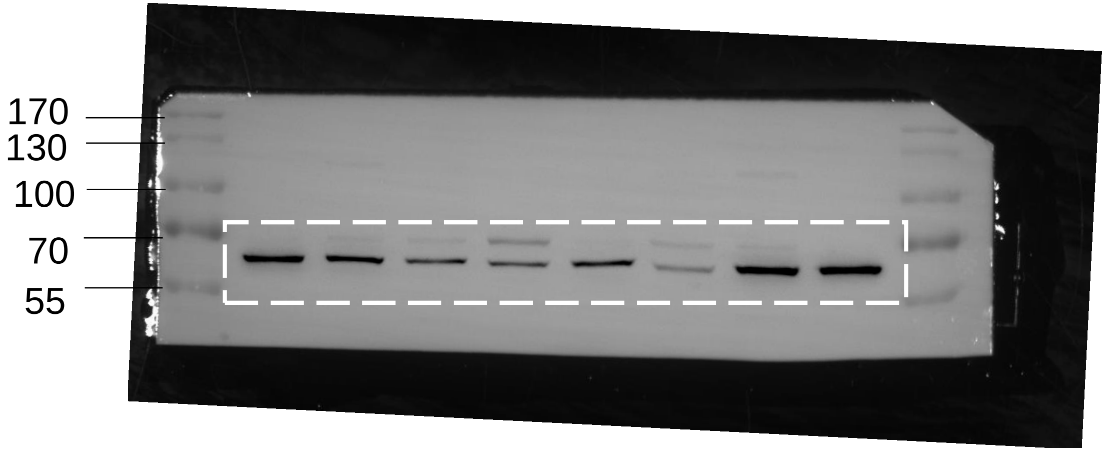

β-tubulin

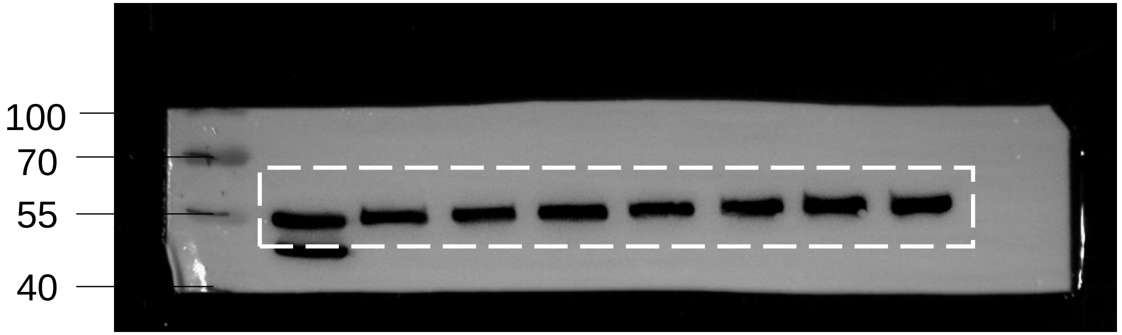

Fig 2A

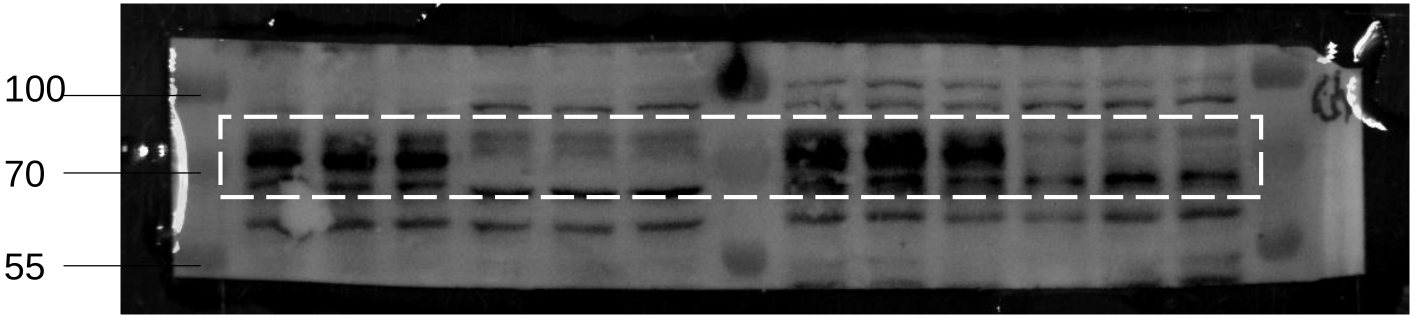

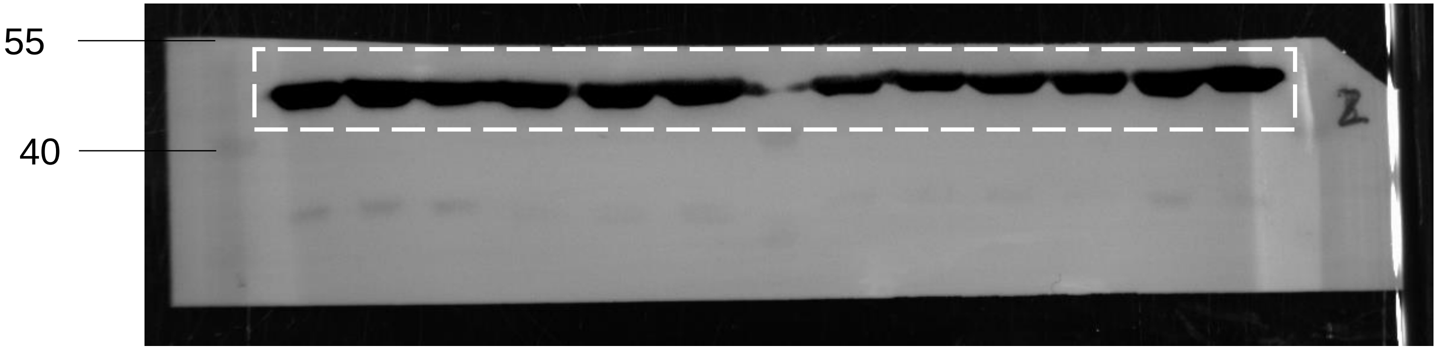

Fig 2F (BT549)

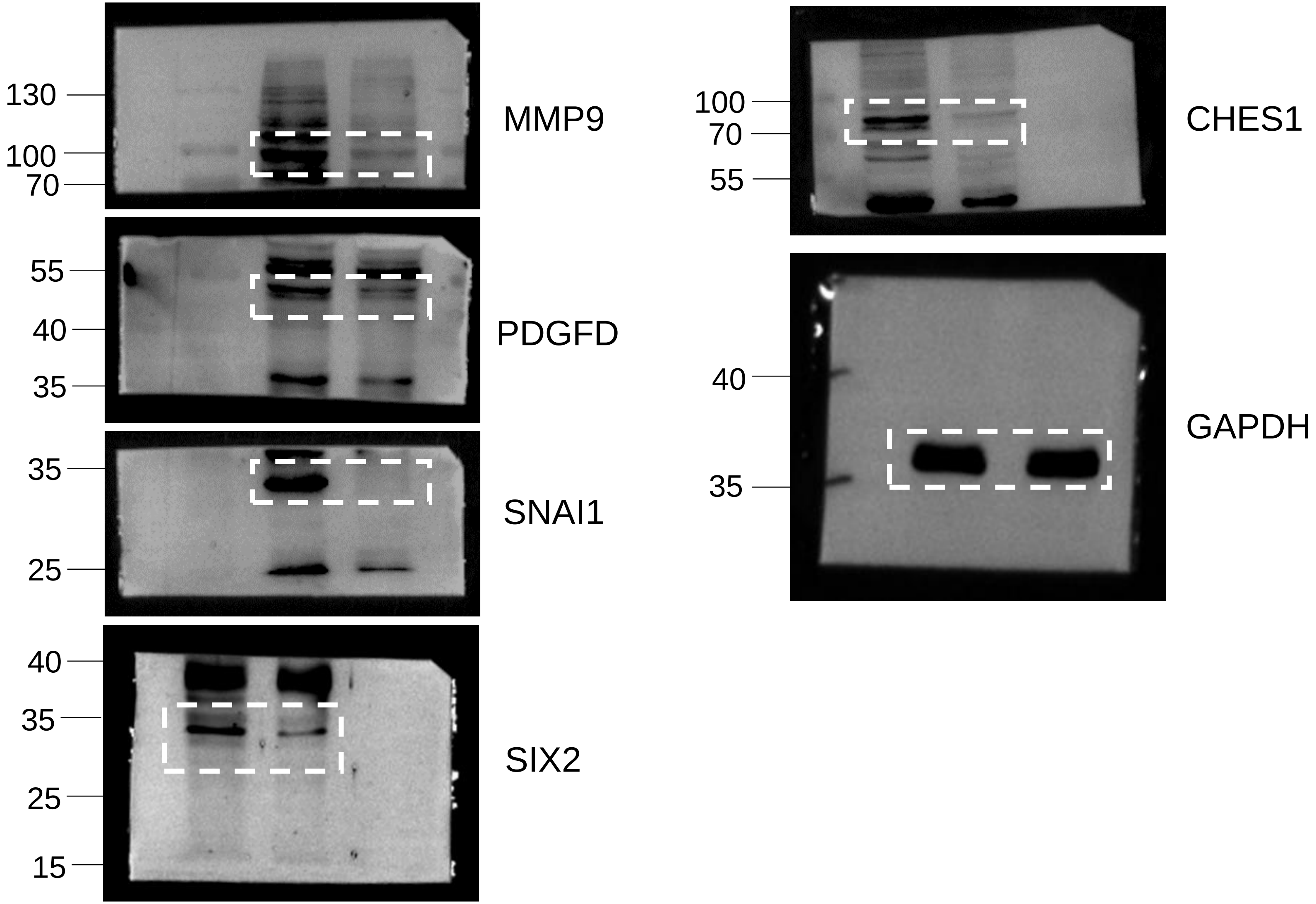
MMP9
130
100
70
PDGFD
55
40
35
SNAI1
35
25
SIX2
40
35
25
15
CHES1
100
70
55
GAPDH
40
35

Fig 2F (MDA-MB-231)

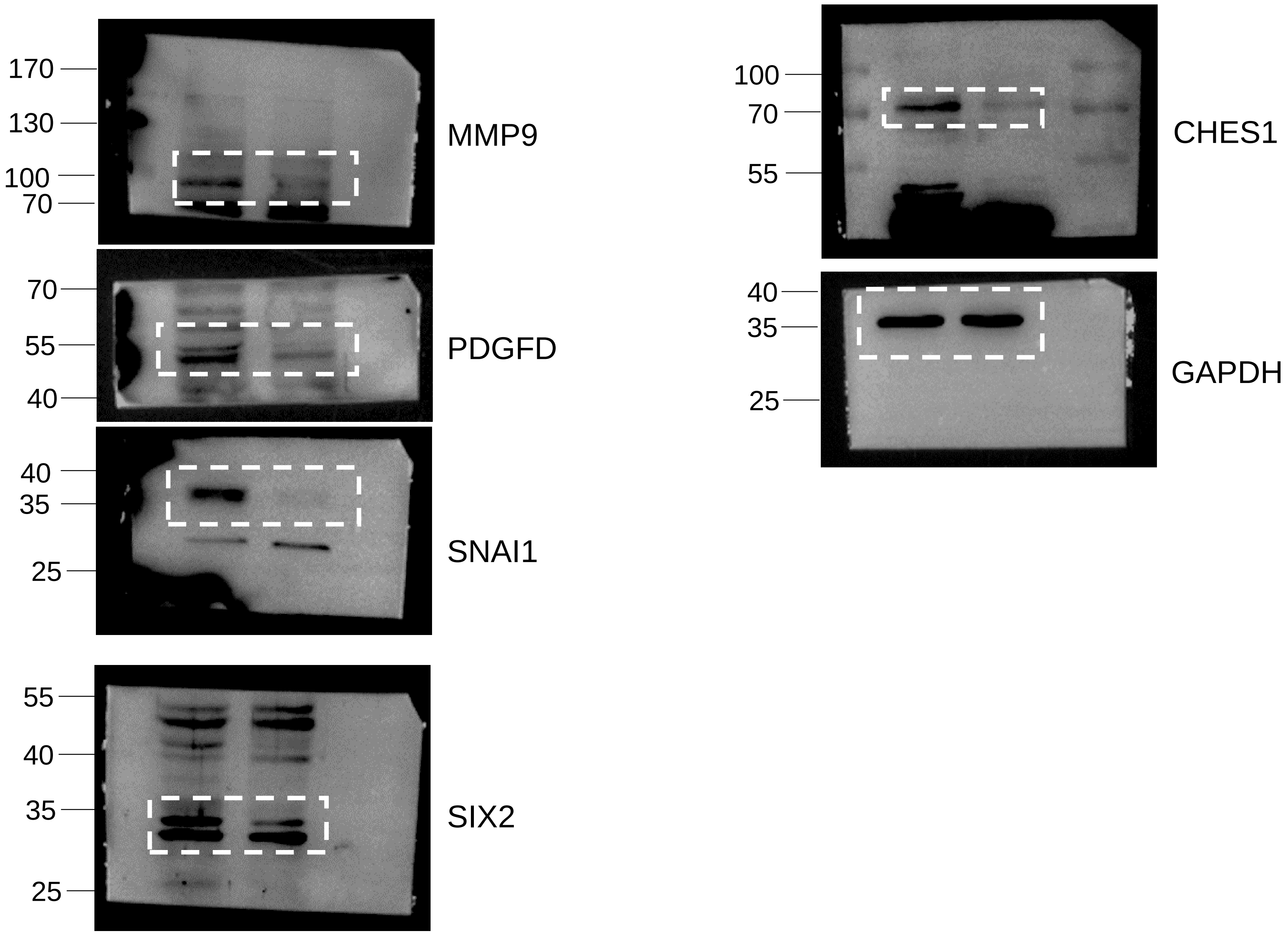

Fig 4A

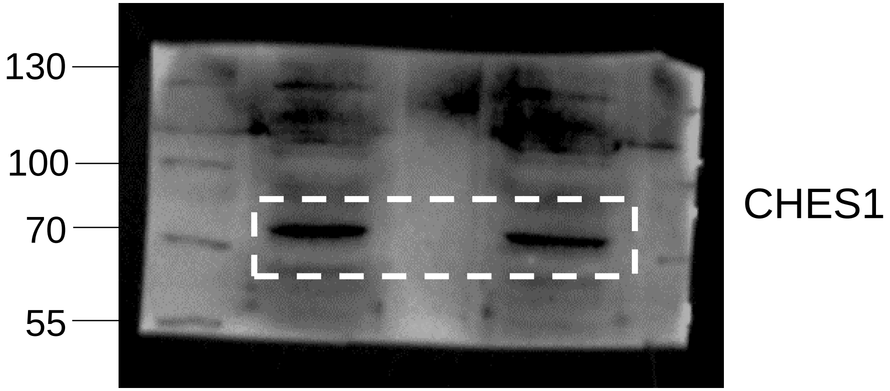

Fig 4B

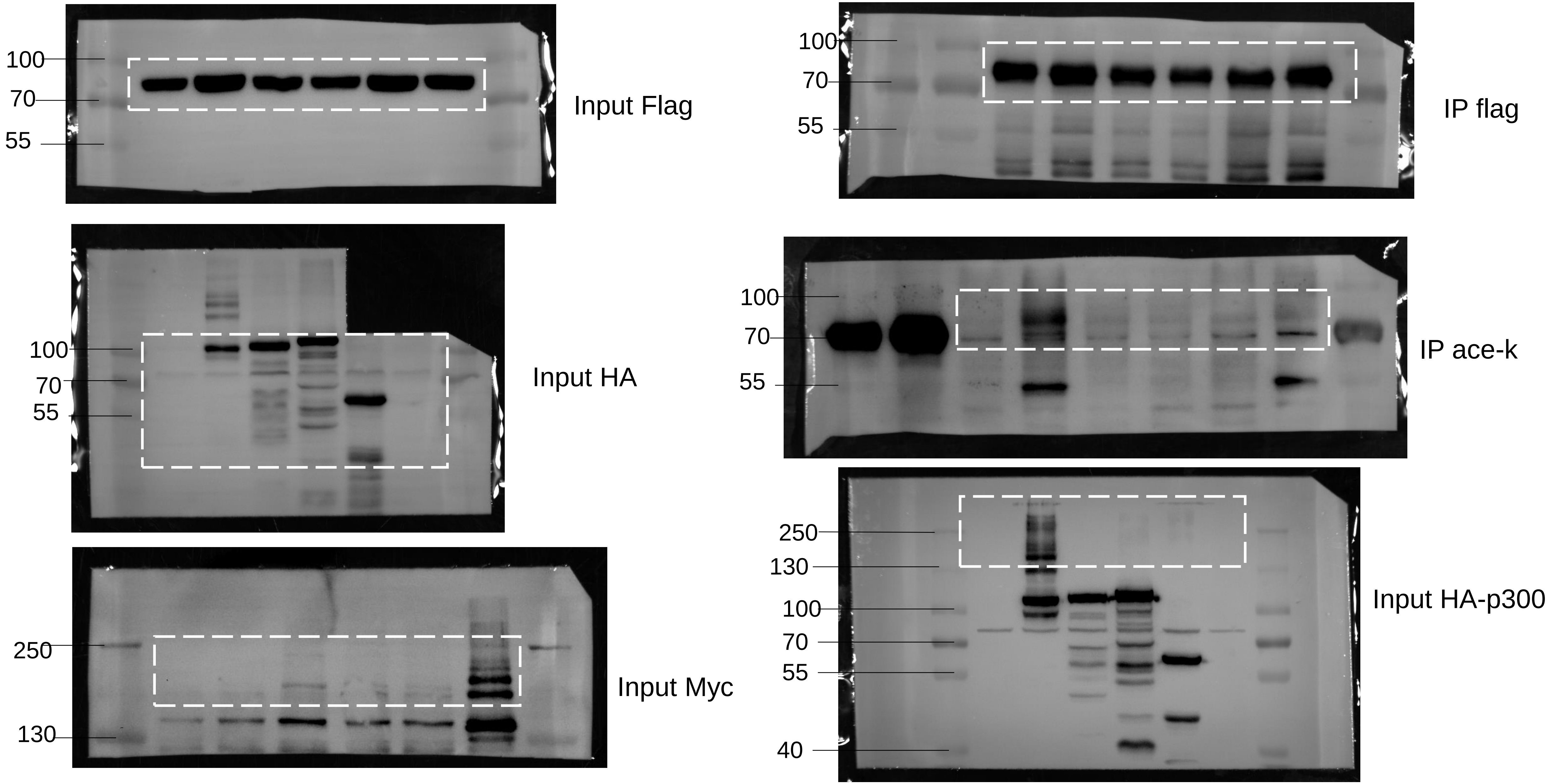
100
70
55
Input Flag
100
70
55
Input HA
250
130
Input Myc
100
70
55
IP flag
100
70
55
IP ace-k
250
130
100
70
55
40
Input HA-p300

Fig 4C

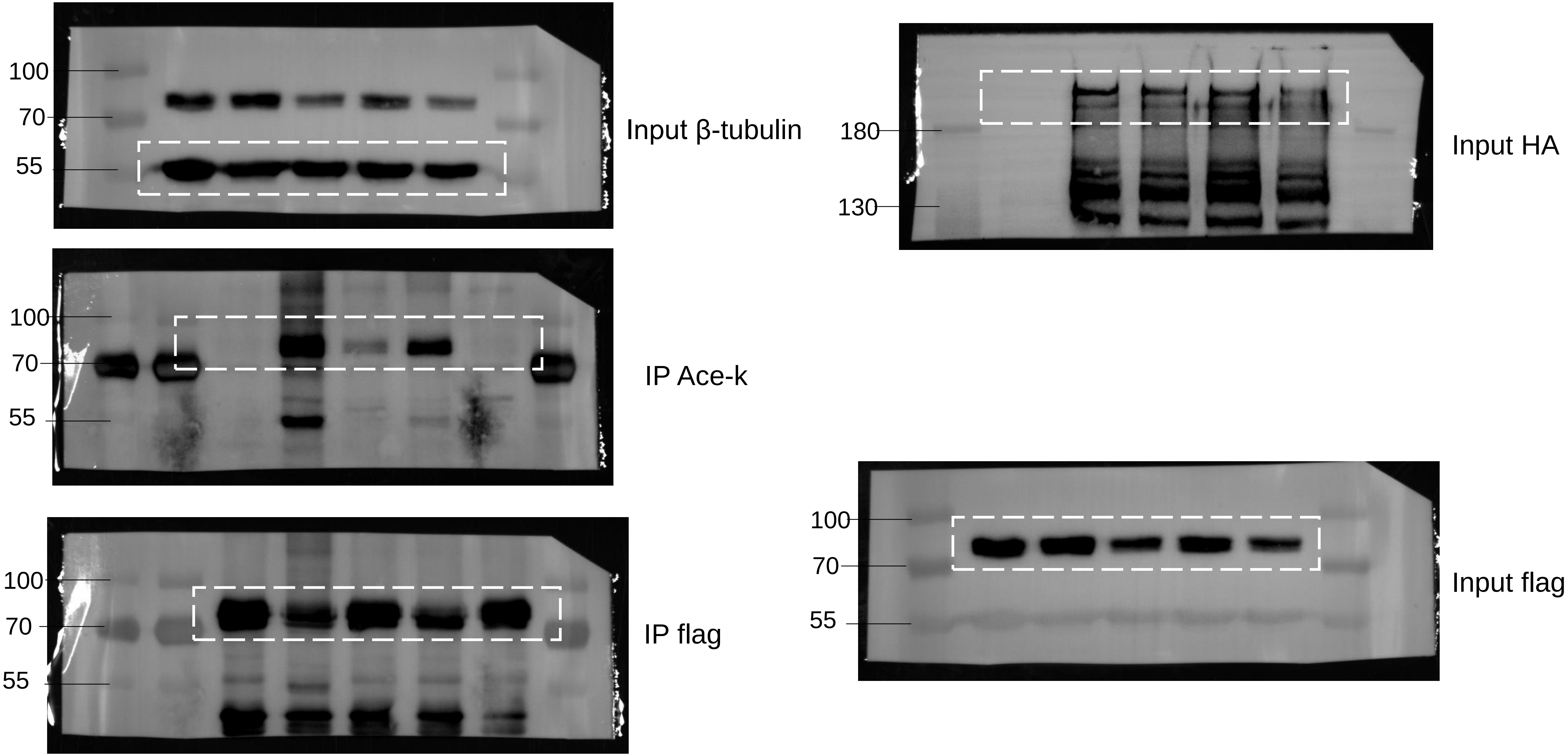
100
70
55
Input β-tubulin
100
70
55
IP Ace-k
100
70
55
IP flag
180
130
Input HA
100
70
55
Input flag

Fig 4D

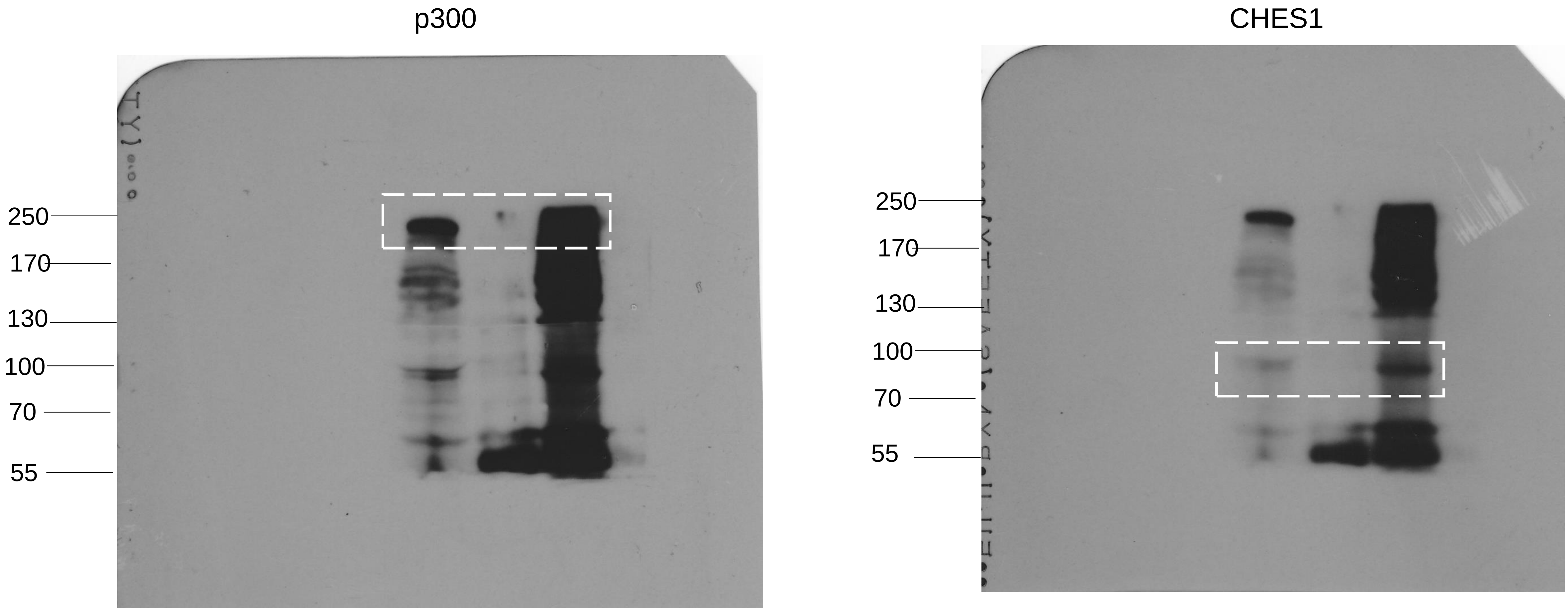

Fig 4E

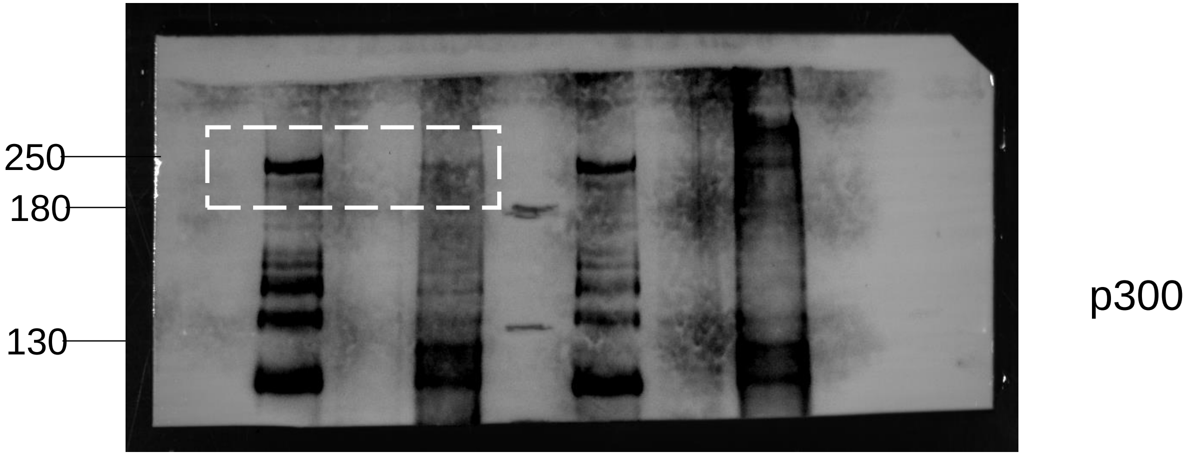

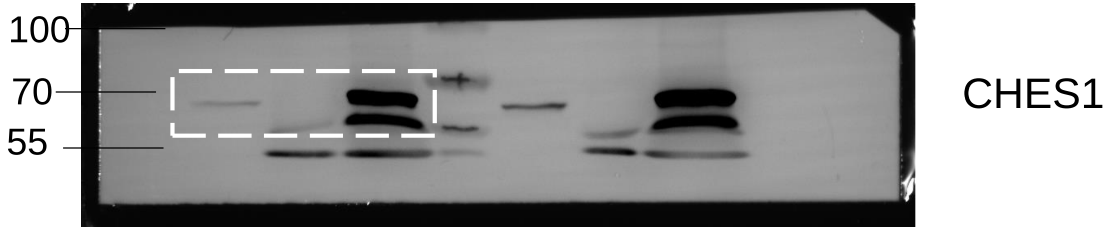

Fig 4F

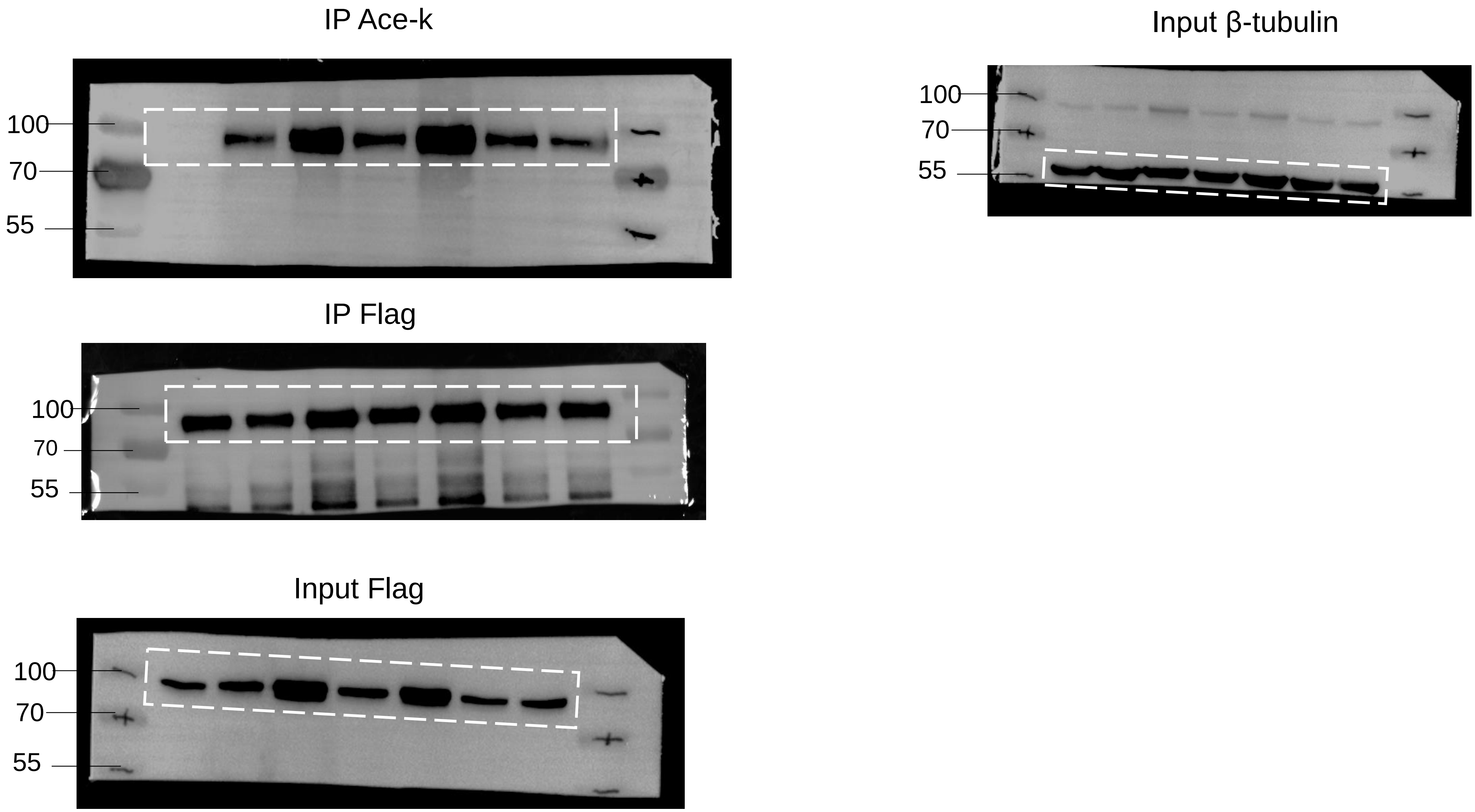
IP Ace-k
100
70
55
IP Flag
100
70
55
Input Flag
100
70
55
Input β-tubulin
100
70
55

Fig 4J

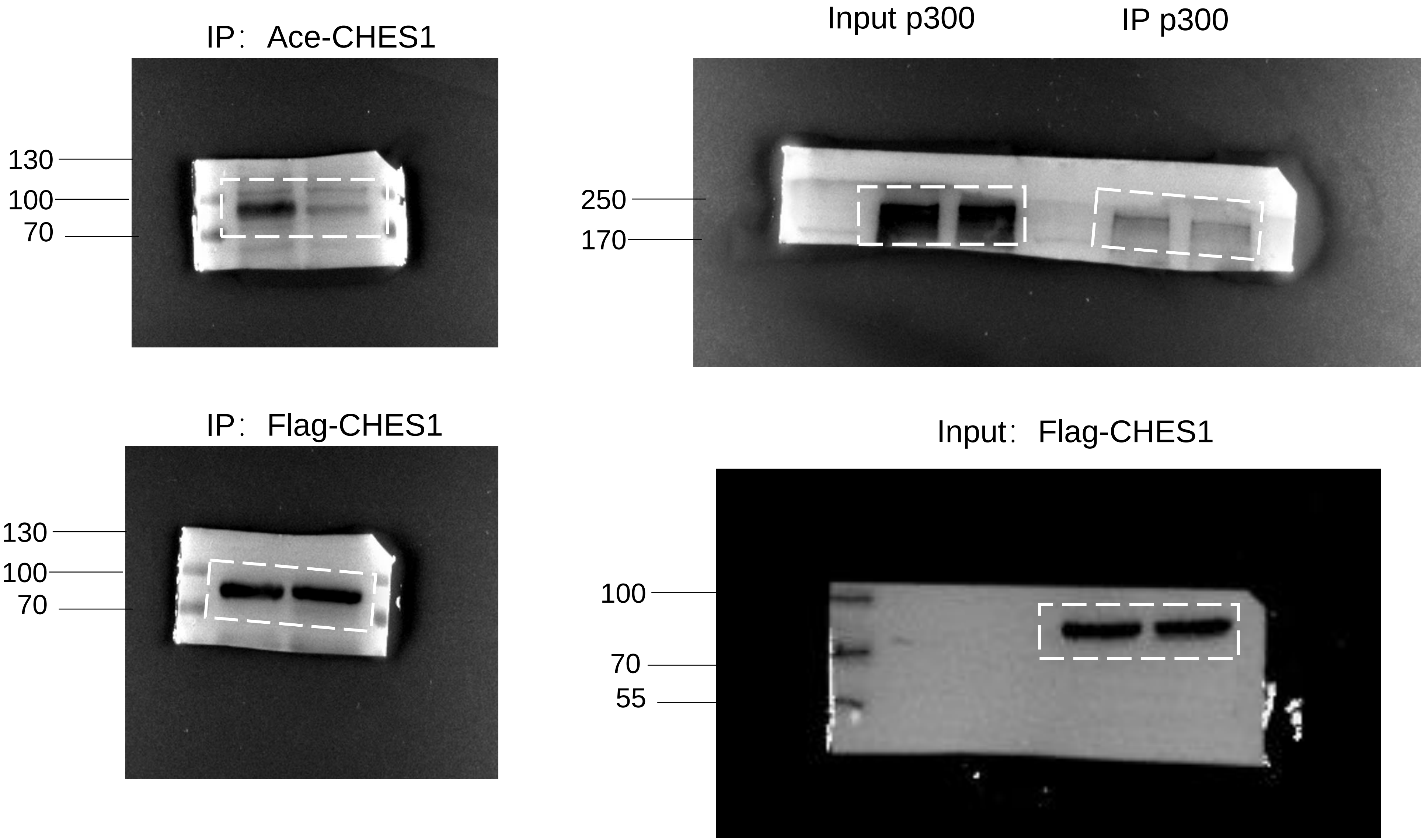
IP: Ace-CHES1
130
100
70
Input p300
IP p300
250
170
IP: Flag-CHES1
130
100
70
Input: Flag-CHES1
100
70
55

Fig 5A

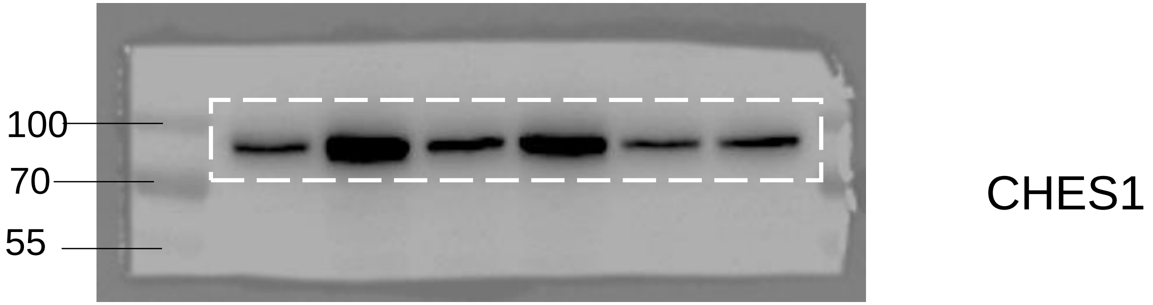

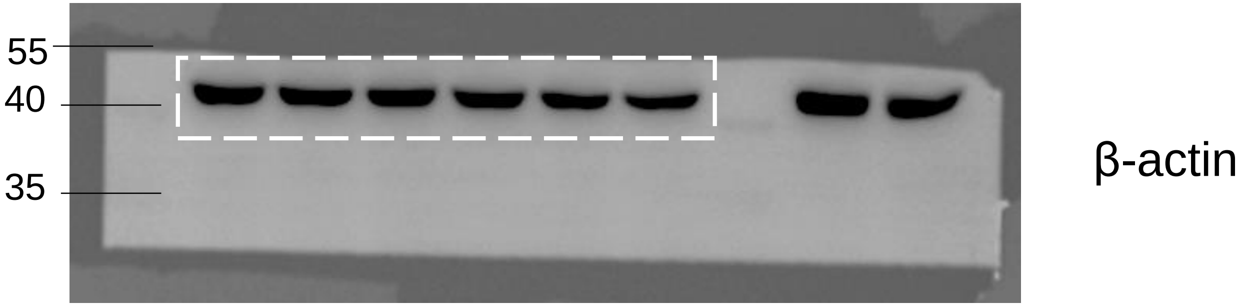

Fig 5B

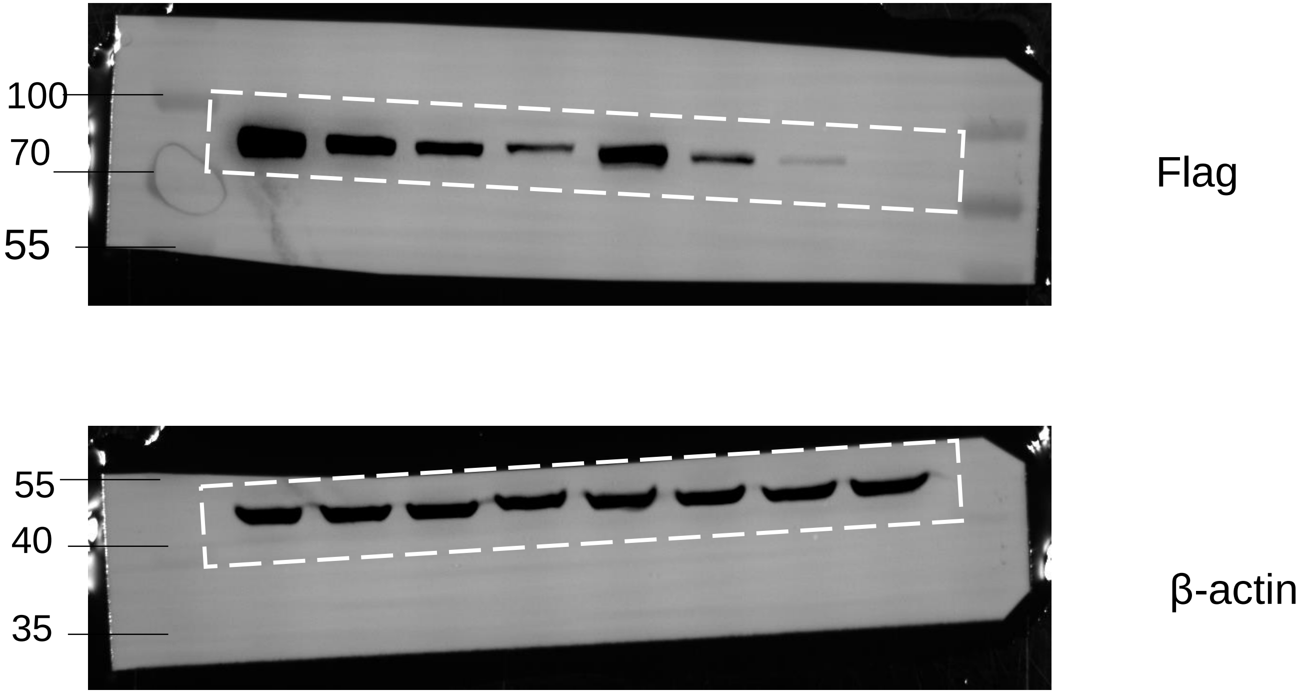

Fig 5C

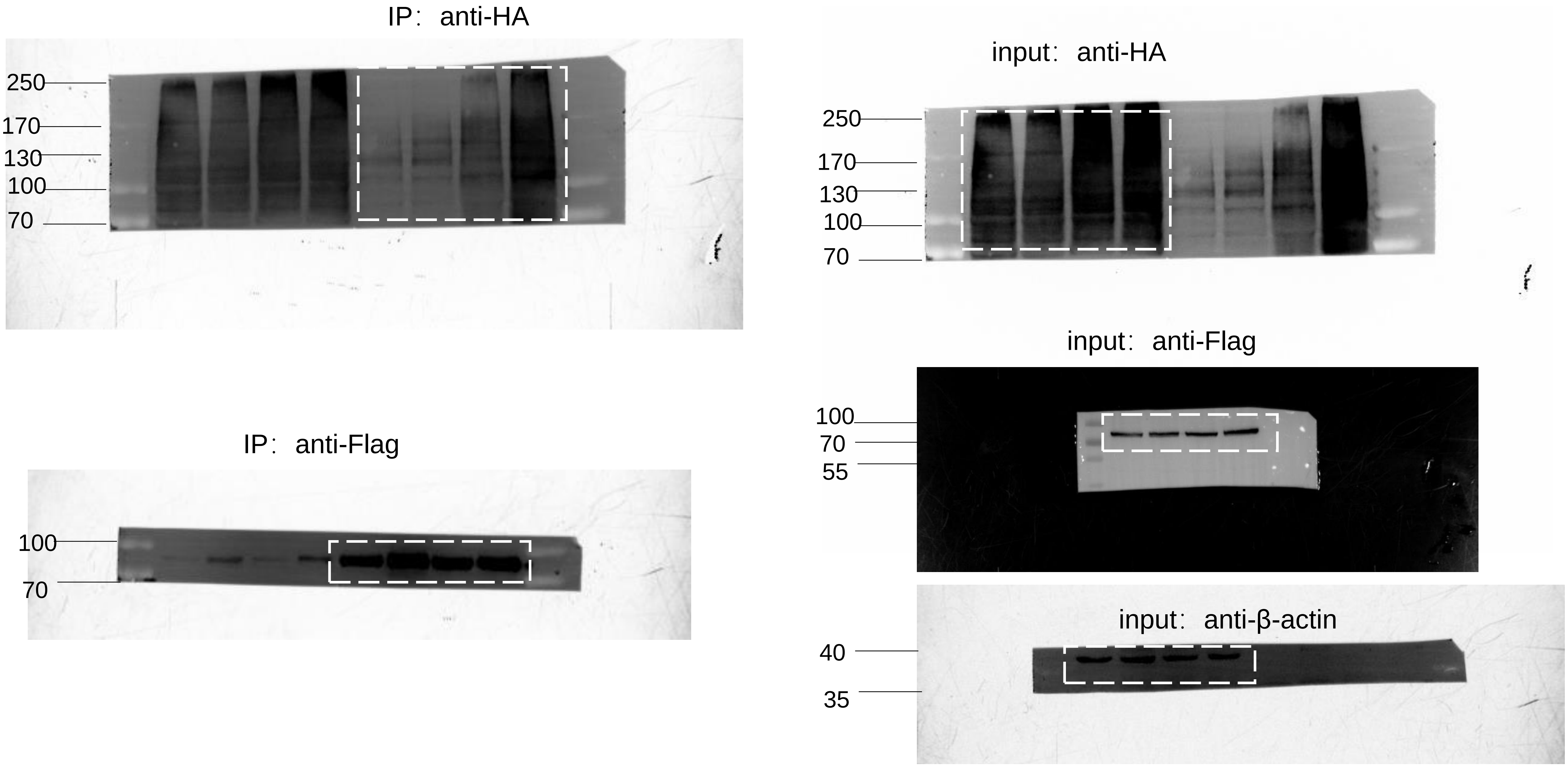
IP：anti-HA
250
170
130
100
70
input：anti-HA
250
170
130
100
70
input：anti-Flag
100
70
55
IP：anti-Flag
100
70
input：anti-β-actin
40
35

Fig 5D

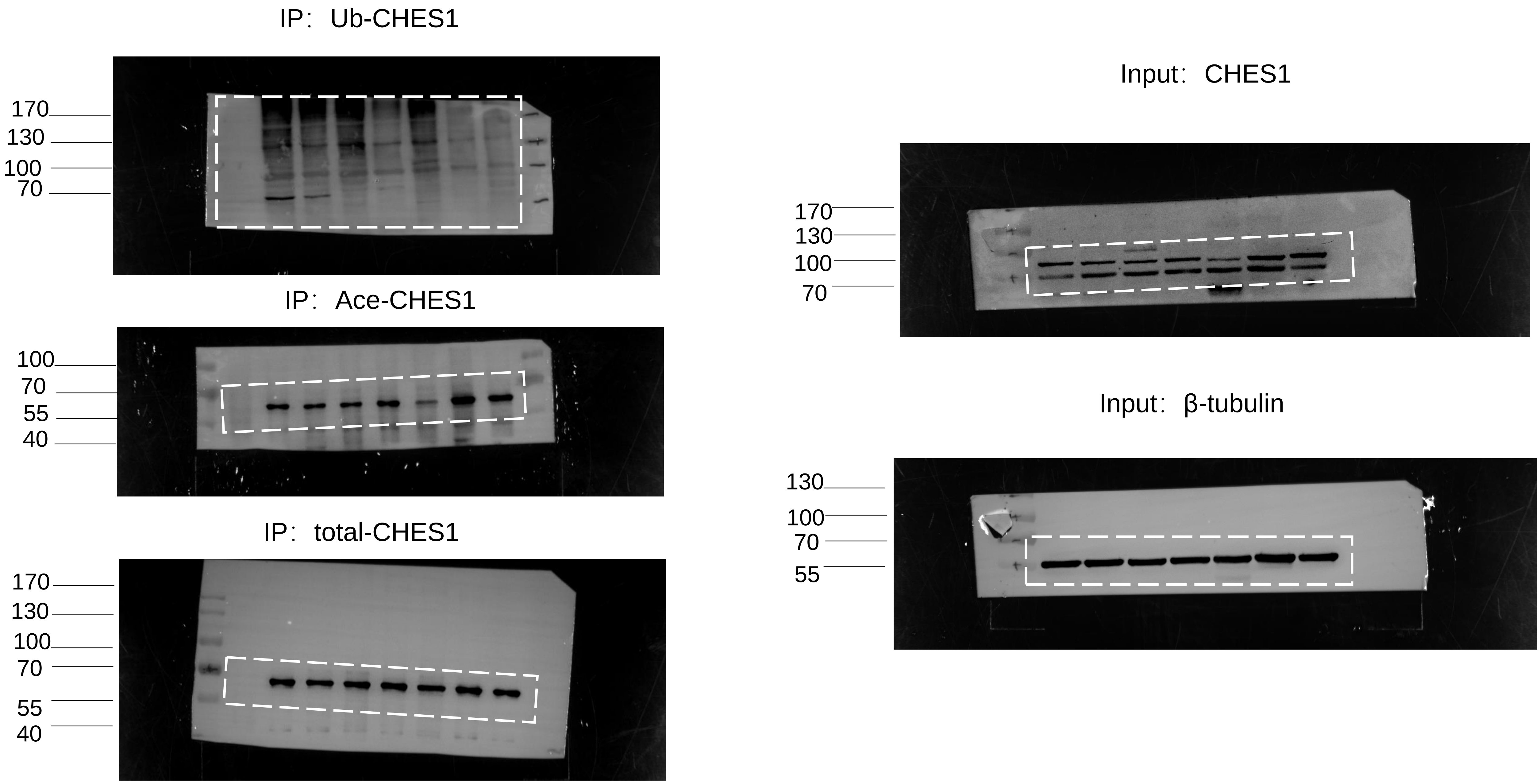
IP：Ub-CHES1
170
130
100
70
IP：Ace-CHES1
100
70
55
40
IP：total-CHES1
170
130
100
70
55
40
Input：CHES1
170
130
100
70
Input：β-tubulin
130
100
70
55

Fig 5E

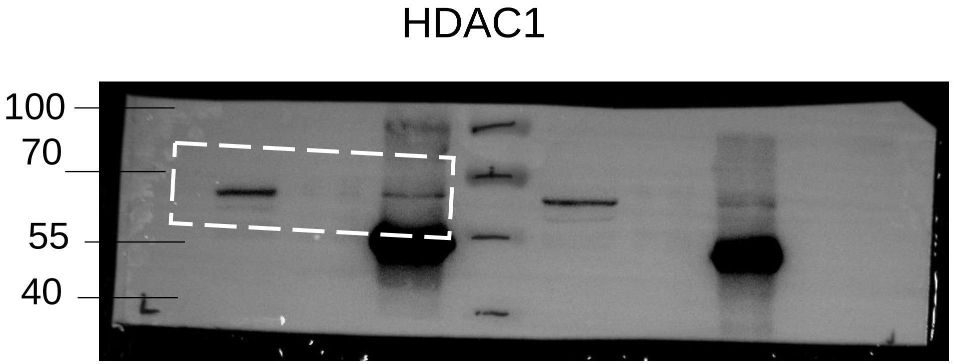

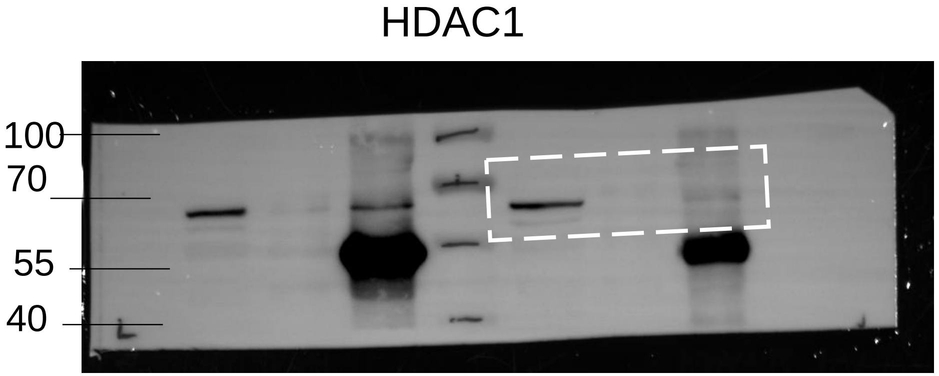

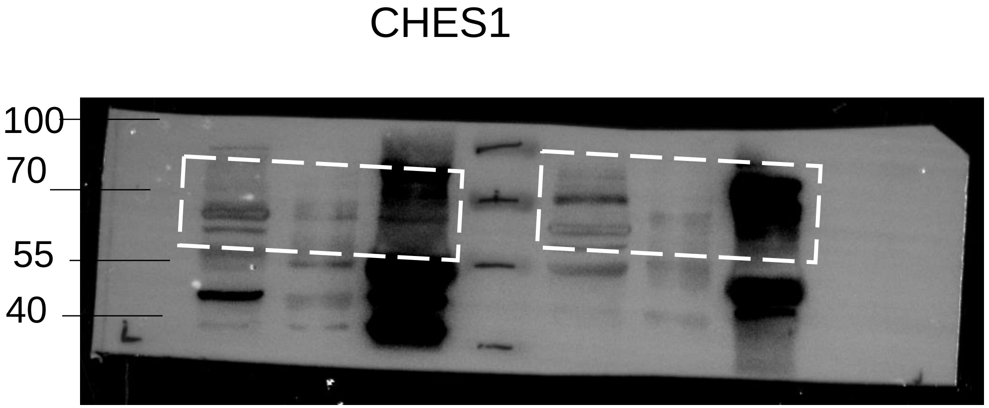

Fig 6E BT549

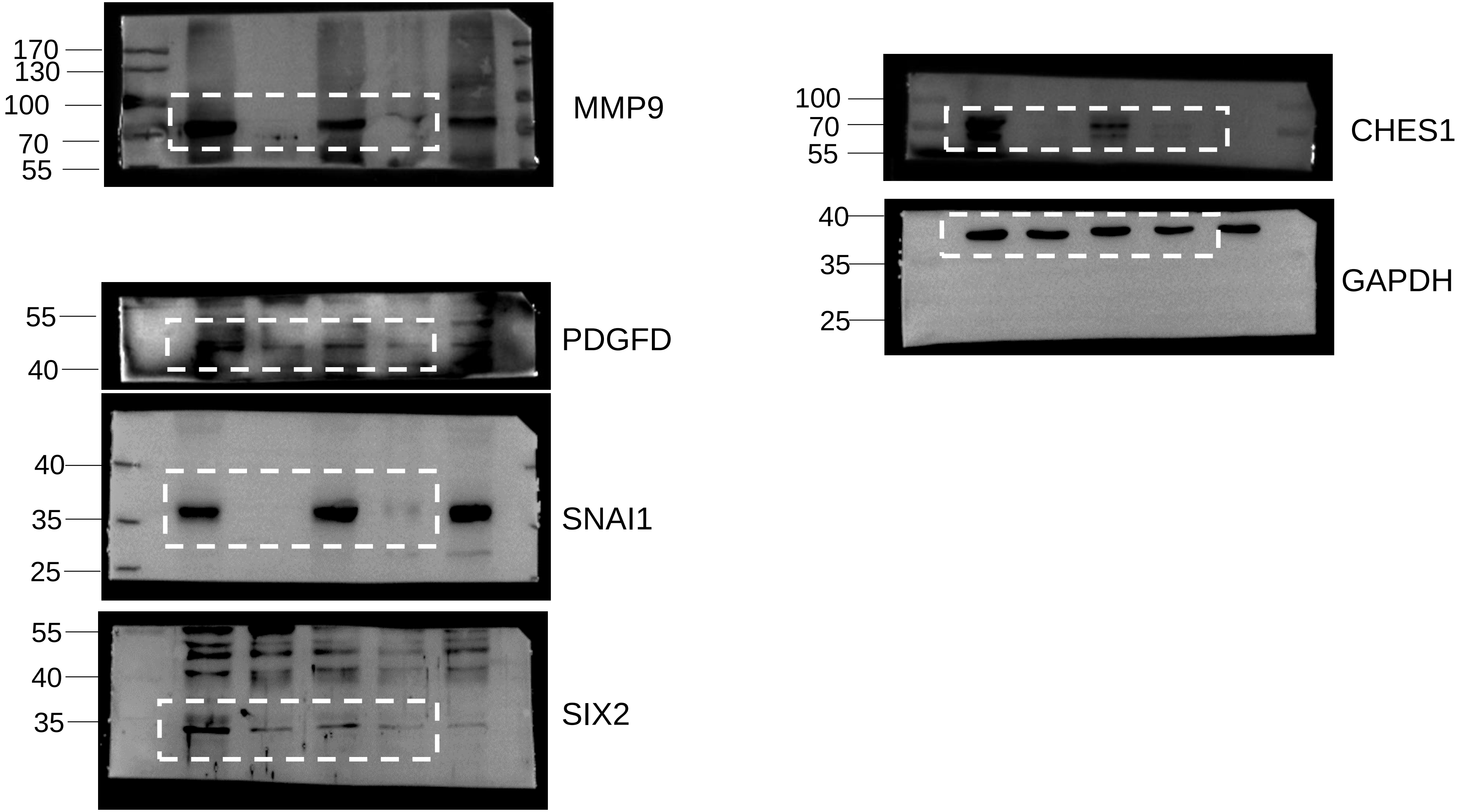
170
130
100
70
55
MMP9
55
40
PDGFD
40
35
25
SNAI1
55
40
35
SIX2
100
70
55
CHES1
40
35
25
GAPDH

Fig 6E MDA-MB-231

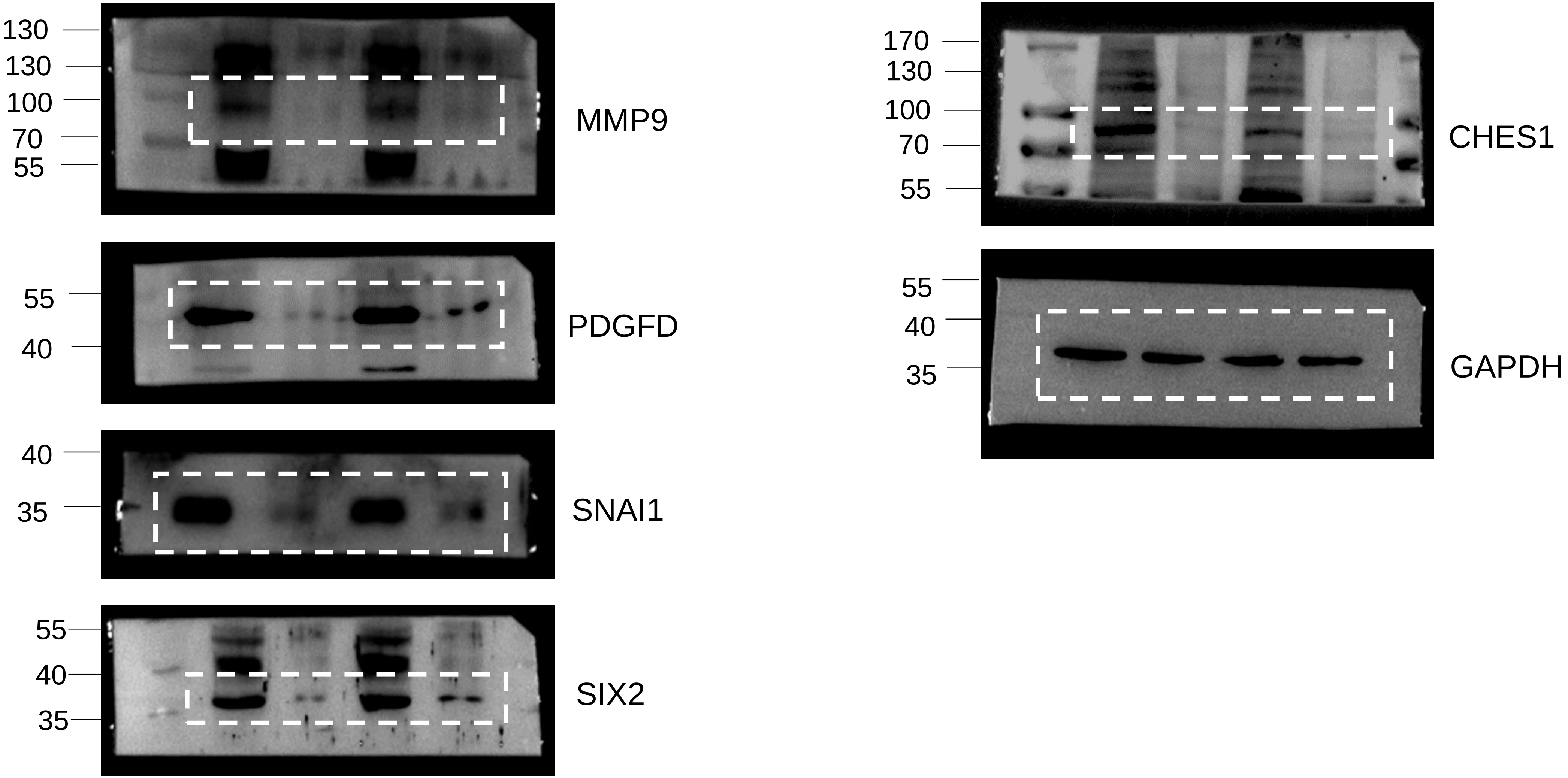

Fig S1B

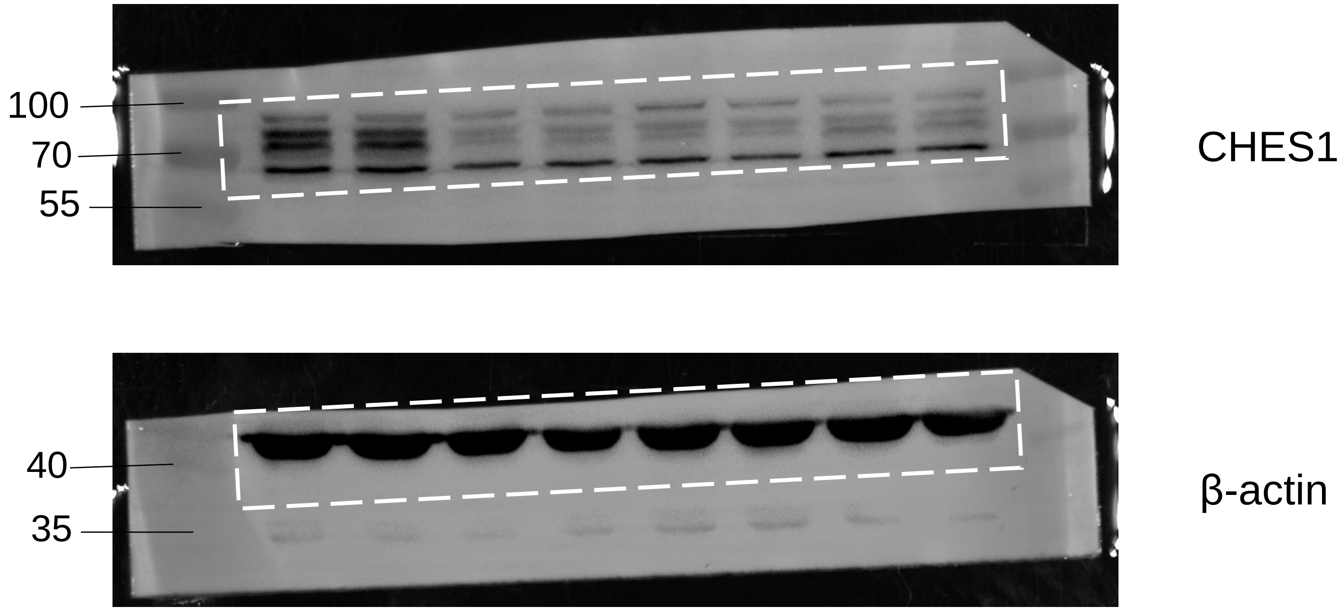

Fig S4A

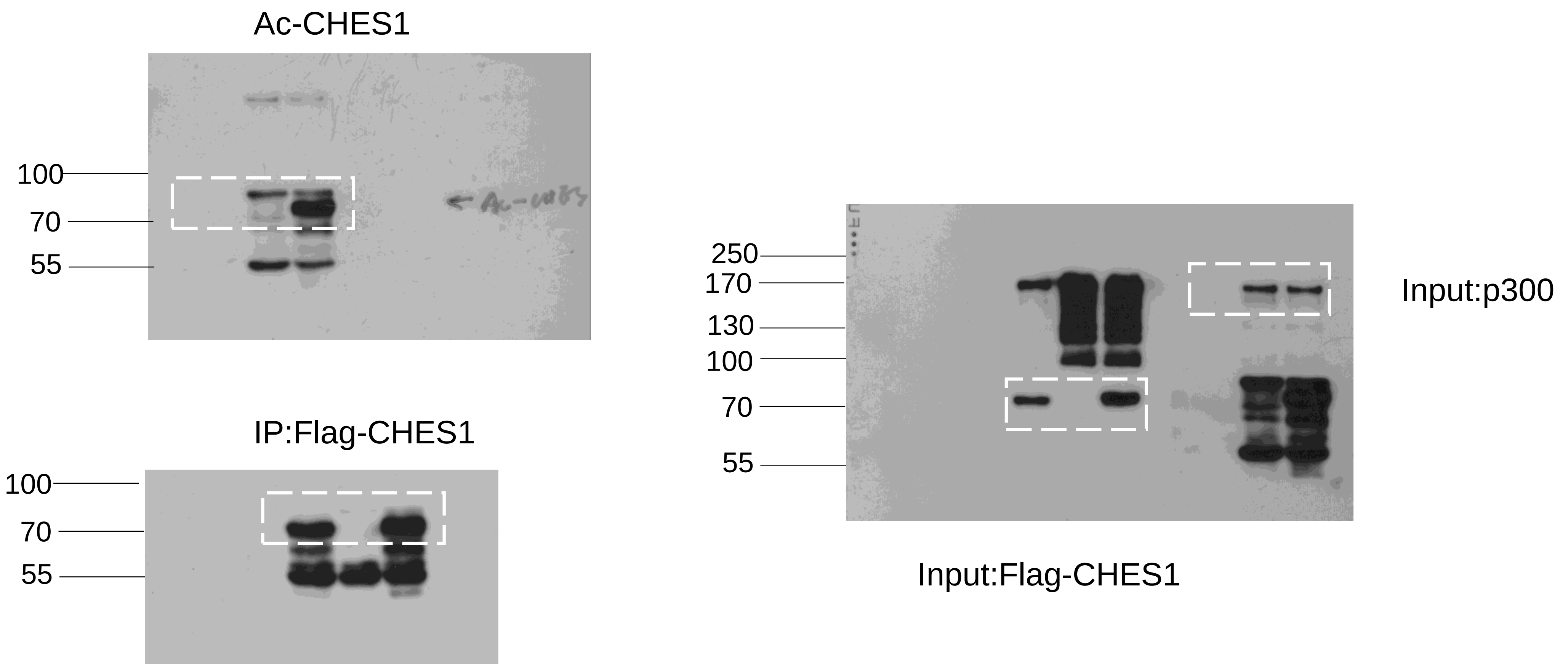
Ac-CHES1
100
70
55
IP:Flag-CHES1
100
70
55
250
170
130
100
70
55
Input:p300
Input:Flag-CHES1

Fig S4B

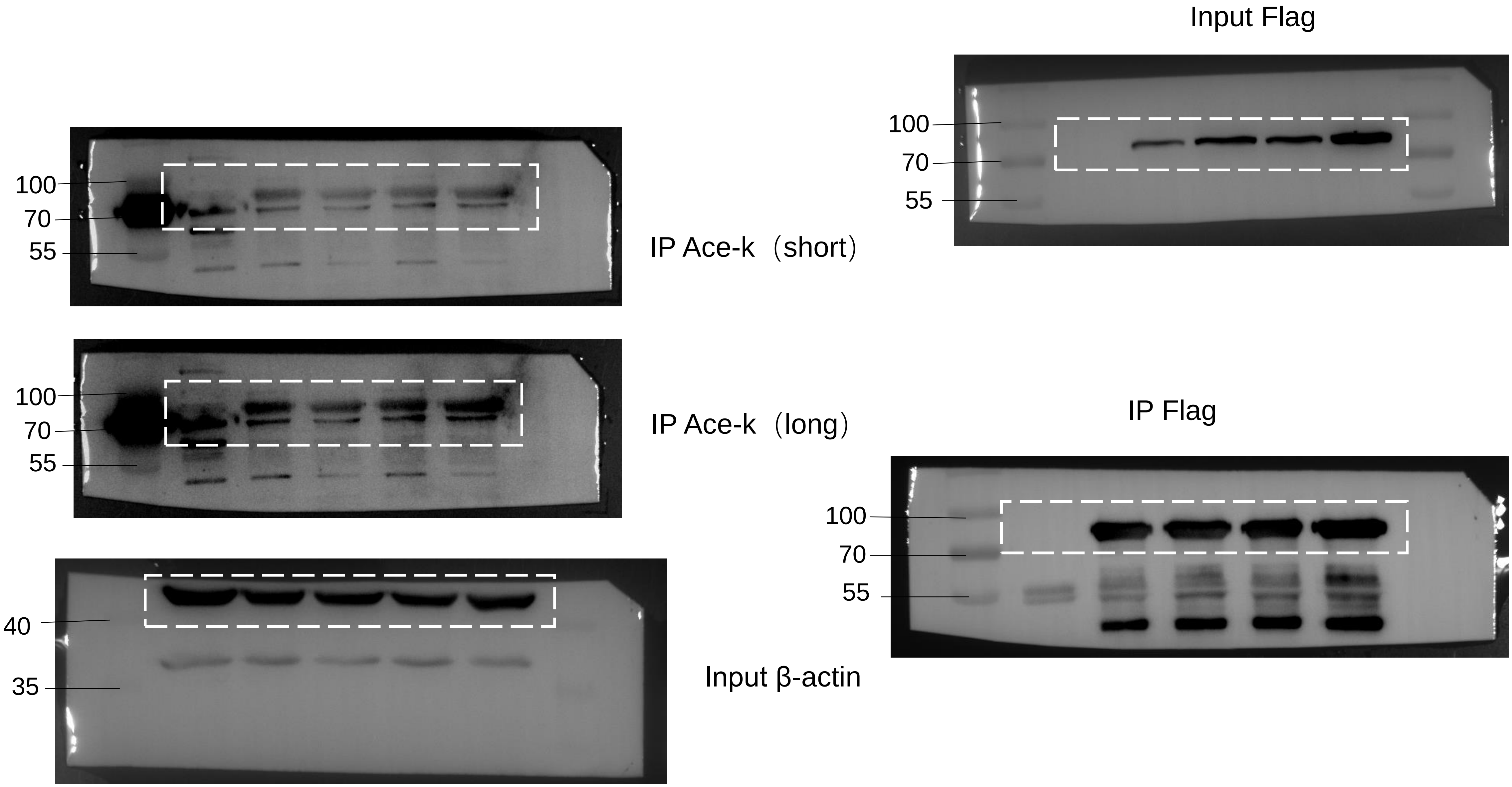

Input Flag
100
70
55
IP Ace-k（short）
100
70
55
IP Ace-k（long）
100
70
55
IP Flag
100
70
55
40
35
Input β-actin

Fig S4C

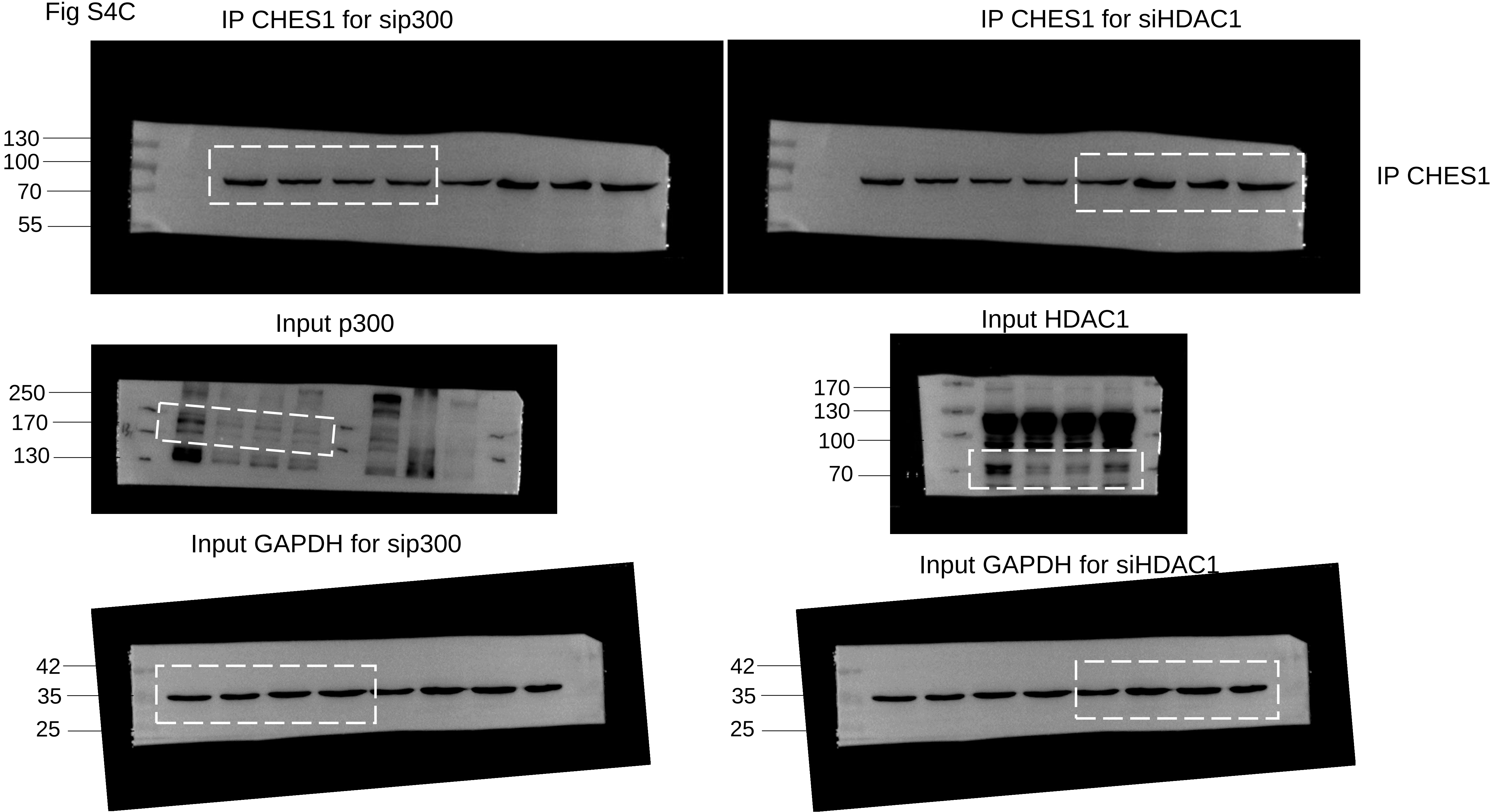
IP CHES1 for sip300
IP CHES1 for siHDAC1
130
100
70
55
IP CHES1
Input p300
250
170
130
Input HDAC1
170
130
100
70
Input GAPDH for sip300
42
35
25
Input GAPDH for siHDAC1
42
35
25

Fig S4D

Input-GFP

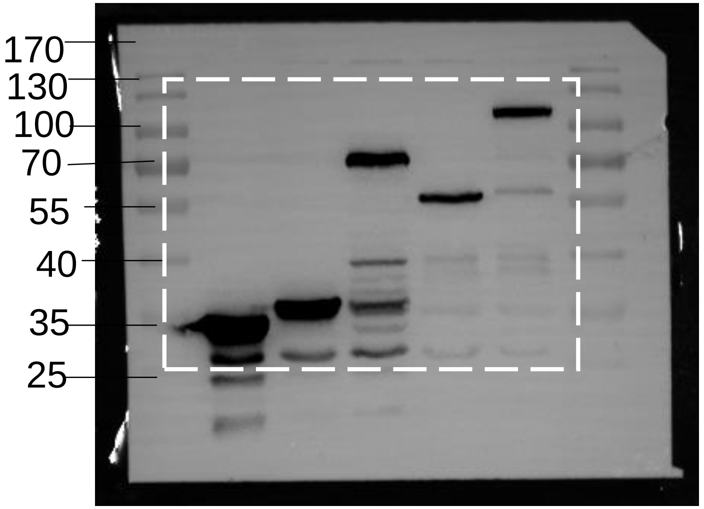

IP-ace-CHES1

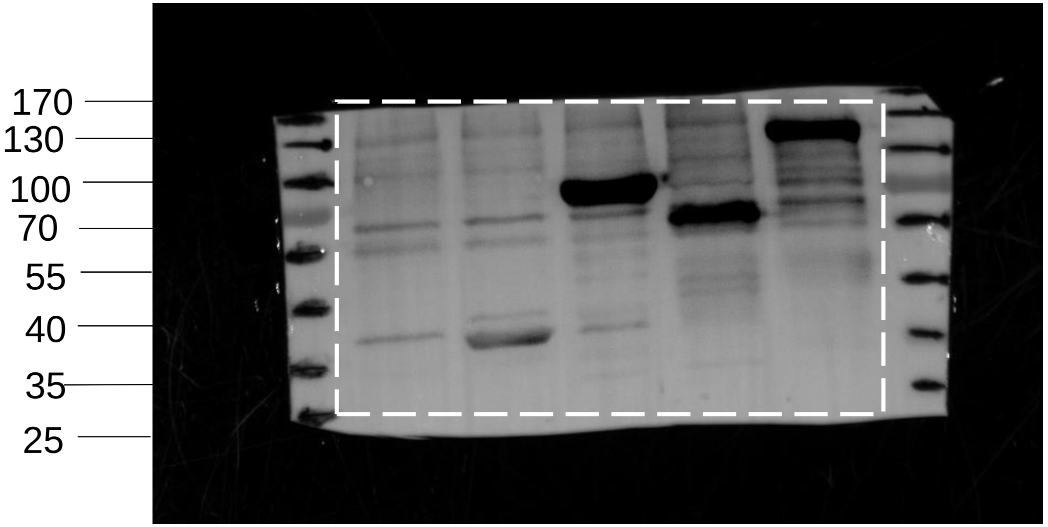

IP: GFP-CHES1

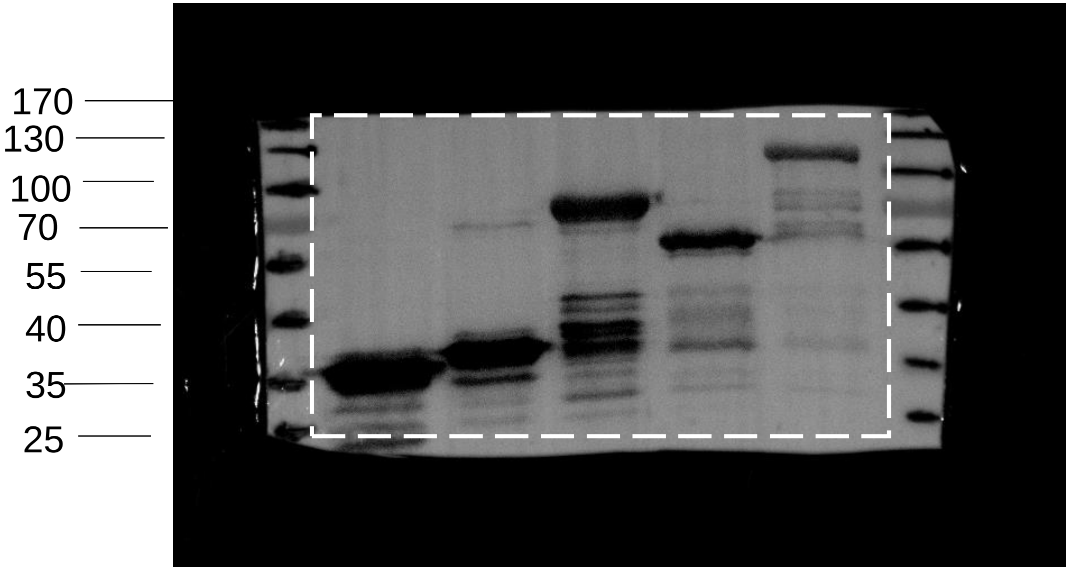

Input-p300

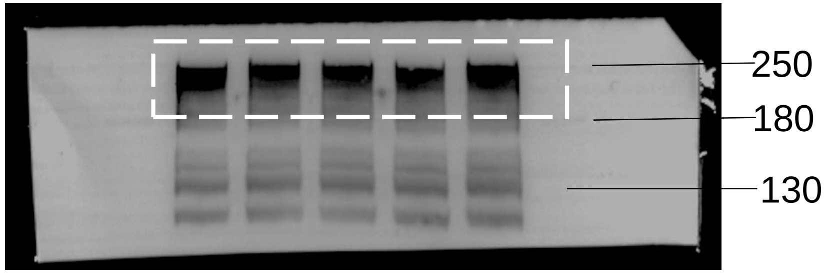

Fig S5A

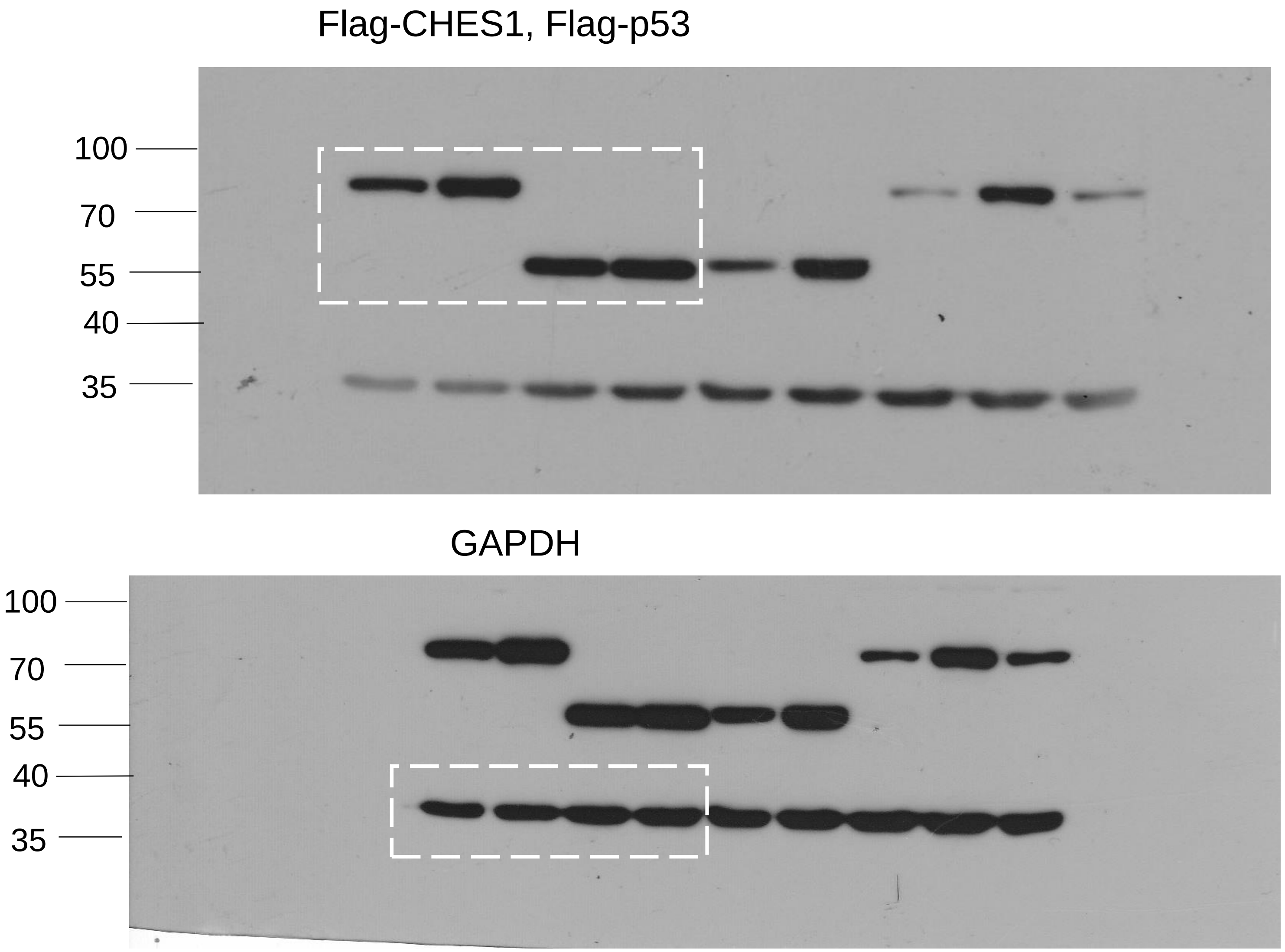

Fig S5C

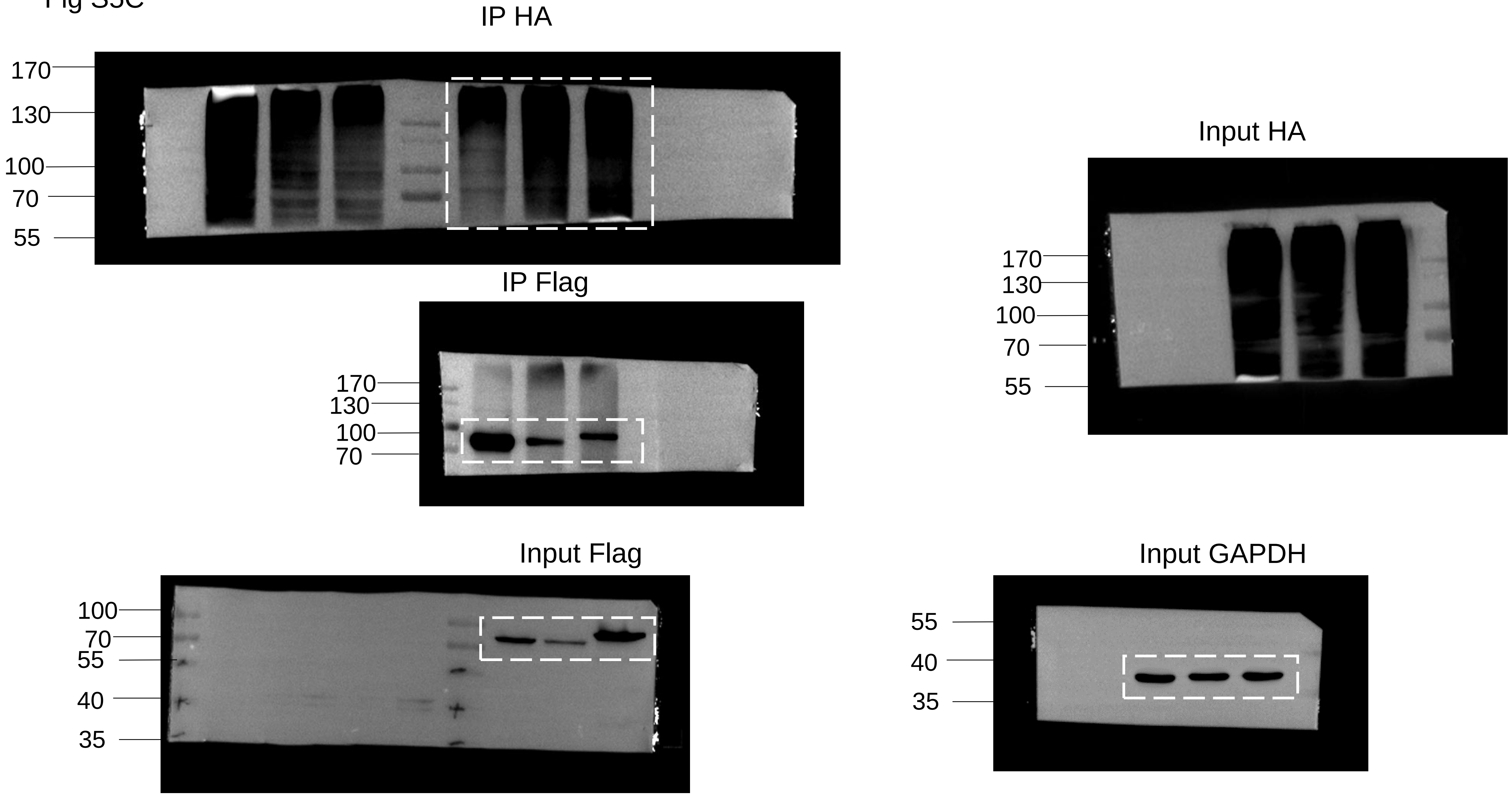
IP HA
170
130
100
70
55
IP Flag
170
130
100
70
Input HA
170
130
100
70
55
Input Flag
100
70
55
40
35
Input GAPDH
55
40
35

Fig S5D

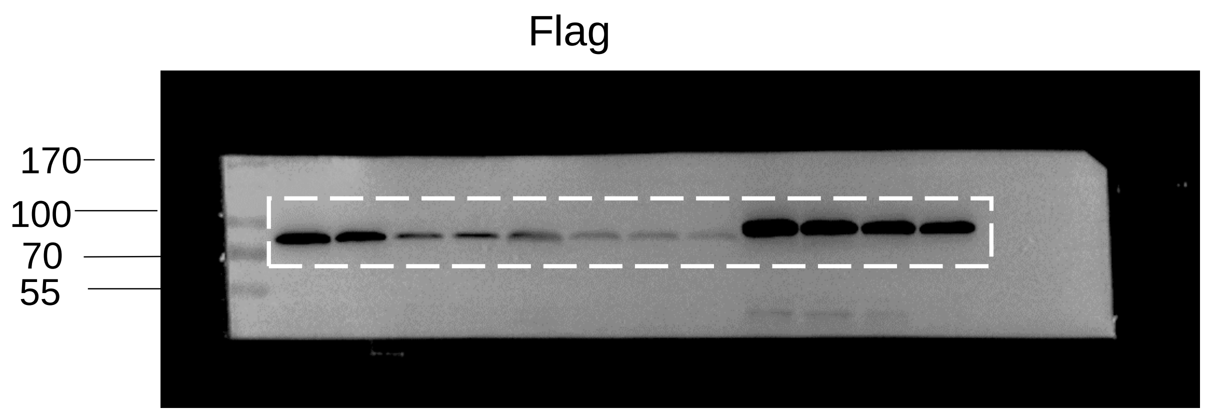

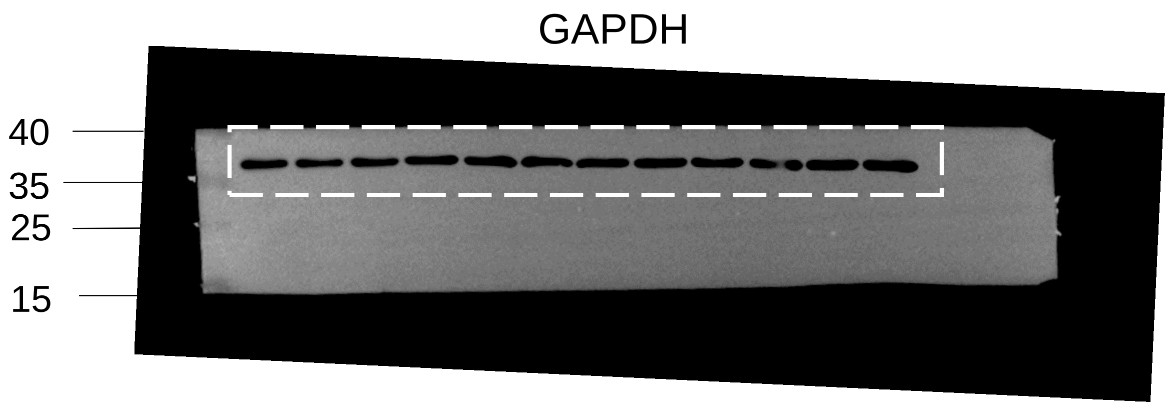

Fig S5E

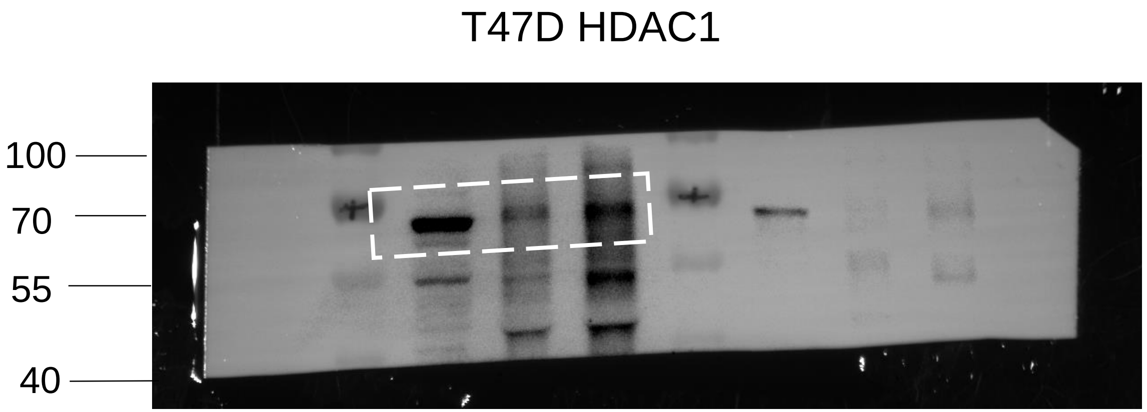
T47D HDAC1
100
70
55
40

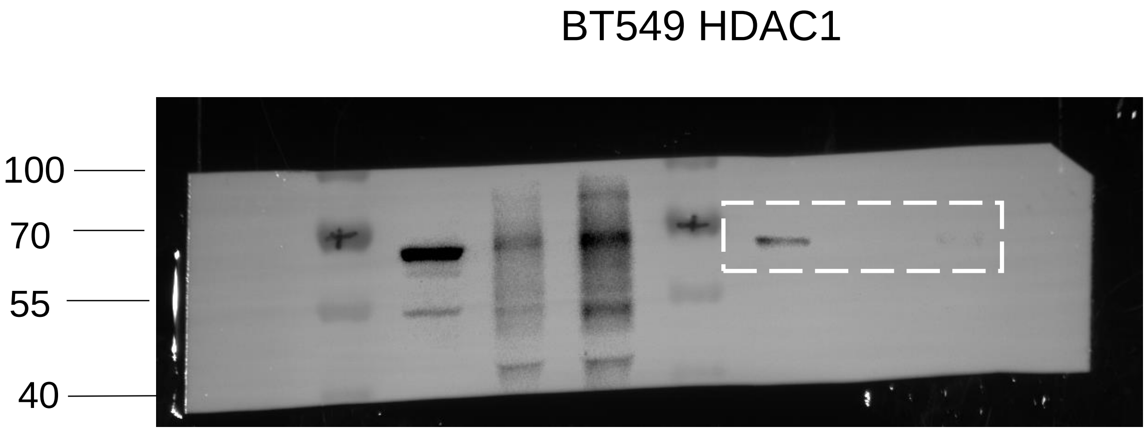
BT549 HDAC1
100
70
55
40

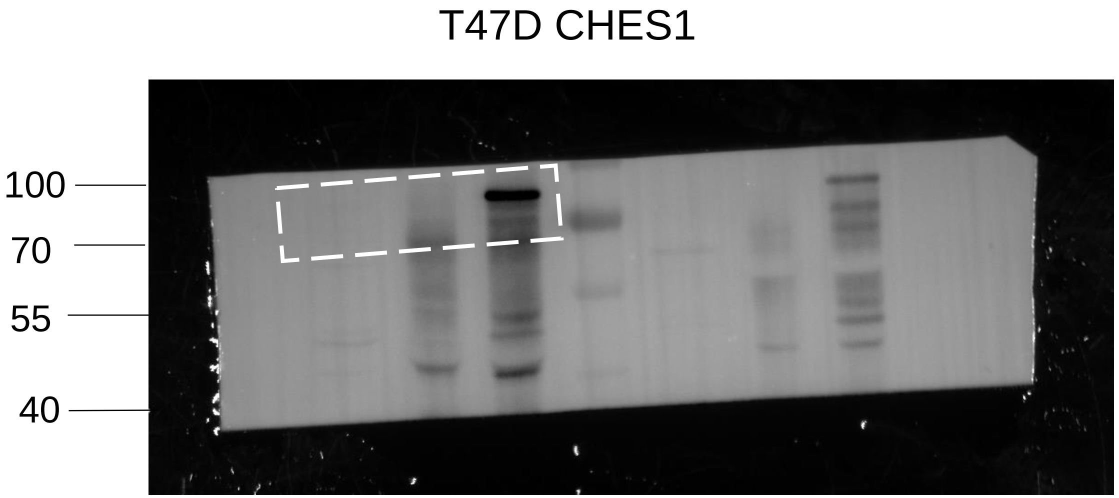
T47D CHES1
100
70
55
40

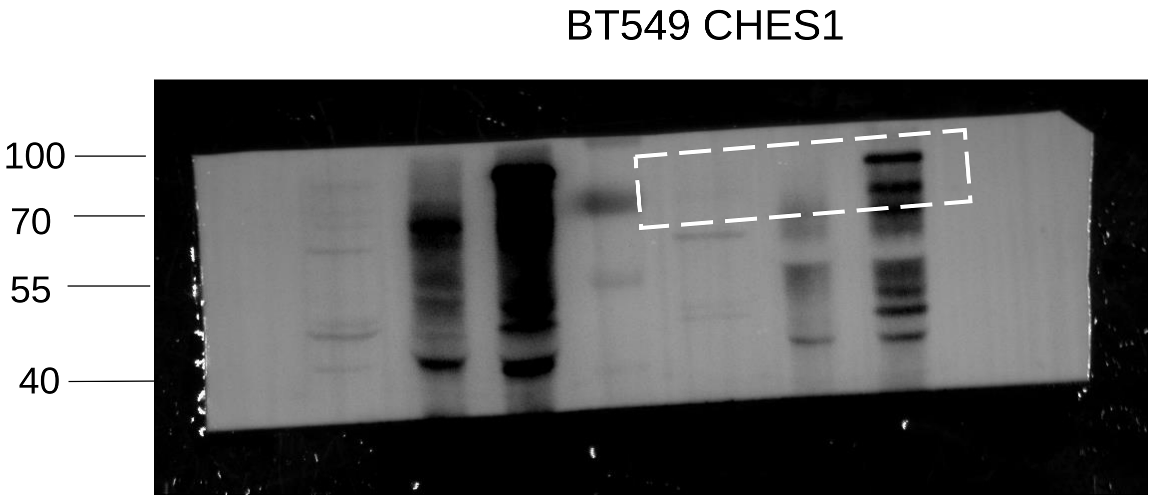
BT549 CHES1
100
70
55
40

Fig S5F

p300

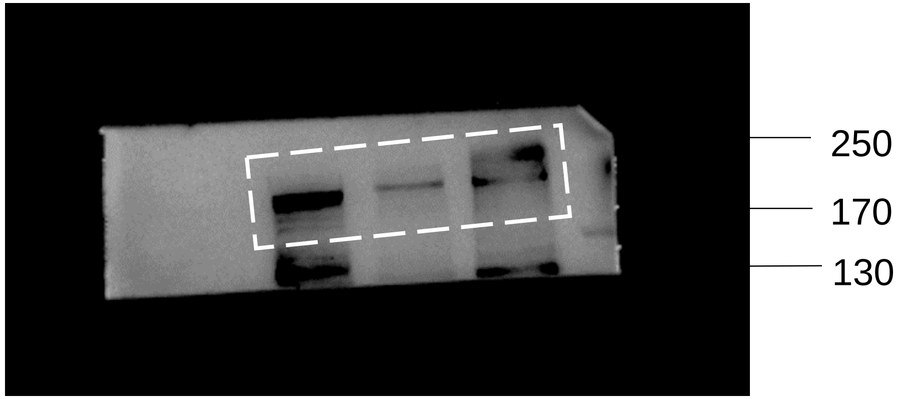

CHES1

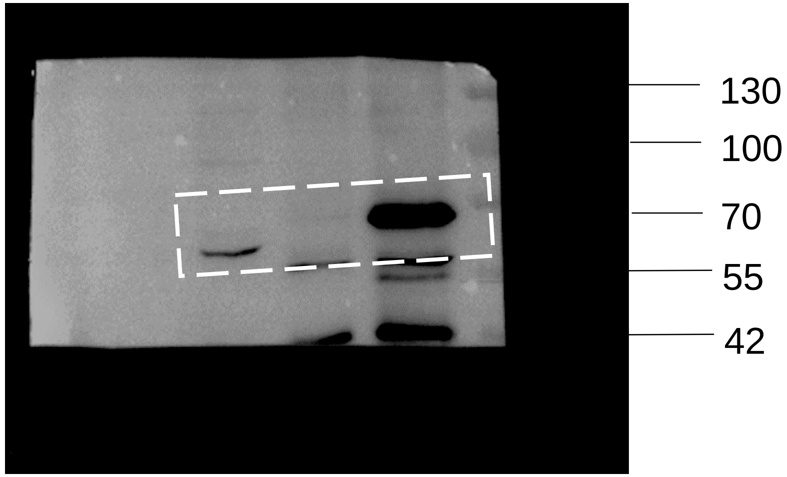

Supplement: Supplementary file 4 — Original Data File [file 41420_2022_1269_MOESM4_ESM.pdf]
